# The Relationship between the Outdoor School Violence Distribution and the Outdoor Campus Environment: An Empirical Study from China

**DOI:** 10.3390/ijerph19137613

**Published:** 2022-06-22

**Authors:** Xidong Ma, Zhihao Zhang, Xiaojiao Li, Yan Li

**Affiliations:** 1 School of Architecture, Tianjin University, Tianjin 300072, China; mxd2813@tju.edu.cn (X.M.); lxj393@tju.edu.cn (X.L.); 2 Key Laboratory of Department of Culture and Tourism of Information Technology of Architectural Heritage Inheritance, Tianjin 300072, China; 3 College of Architecture and Urban Planning, Tongji University, Shanghai 200082, China; zhang_zhihao1998@163.com

**Keywords:** outdoor school violence distribution (OSVD), outdoor campus environment (OCE), Spatial Syntax theory, violence type, spatial attribute, regression analysis

## Abstract

It is widely believed that outdoor environmental design contributes to outdoor violence prevention. To enhance the effectiveness of environmental design, the intrinsic link between the outdoor school violence distribution (OSVD) and the outdoor campus environment (OCE) should be fully considered. For this purpose, this study investigated boarding school L, located in southern Zhejiang Province of China, through a questionnaire and Spatial Syntax theory. Based on the questionnaire marker method (*N* = 338, 50.59% female), the OSVD was mapped using the kernel density estimation in ArcGIS, including four types of teacher-student conflict: verbal bullying, physical conflict, and external intrusion. The spatial analysis of the OCE (spatial configuration and spatial visibility) then was generated by the DepthmapX, involving four spatial attributes such as integration, mean depth, connectivity, and visibility connectivity. Statistical analysis results indicated the correlation between the OSVD and both the spatial configuration and spatial visibility of the OCE. For the different violence types, there were differences in the impact relationships, with integration being a significant predictor of teacher-student conflict and physical conflict (*p* < 0.01) and a general predictor of verbal bullying (*p* < 0.05), while mean depth was a significant predictor of physical conflict (*p* < 0.01), but not recommended as a predictor of external intrusion. This study explores and predicts the relationship between the OSVD and the OCE, providing guidance and evidence for school violence prevention environmental design. It is a novel attempt, but still challenging and requires more research to refine.

## 1. Introduction

Although schools are supposed to be harmonious and stable places, there have been many incidents of student violence in recent years which have seriously damaged the campus atmosphere. School violence takes many forms, not just physical conflict, but also social violence such as verbal intimidation, cyber violence, group isolation, and student-teacher conflict [1,2]. The persistence of school violence can influence the order of teaching and learning, the campus living environment, and the healthy development of students in varying degrees, and, in particular can damage students’ physical and mental health [3], reduce subjective well-being [4,5], and cause great psychological disorders [6,7]. Meanwhile, school violence is a global problem with some 246 million children worldwide experiencing different forms of violence and almost a third (32%) of students being bullied by their peers on one or more days, according to the reports that were published by UNESCO [1,8]. In addition, the proportion of bullied students is highest in the Middle East, North Africa, and Sub-Saharan Africa and lowest in Central America, the Caribbean, and Europe. In China, a field study that was conducted by Central China Normal University in six provinces with a sample of more than 10,000 students in more than 130 primary and secondary schools, showed that the school violence rate was 32.4% from 2019 to 2020 [9,10]. It is, therefore, clear that research into the school violence prevention is urgent, especially in developing countries such as China.

In recent years, many scholars around the world have begun to explore the causes of school violence from the perspectives of culturally transformative education [11], adolescent personality traits [12,13], student physicality [14], family background [15], teaching climate [16], and social capital [17] in order to prevent or reduce the occurrence of school violence, and have proposed violence prevention measures such as dialogue models [18], drama therapy programs [19], and community participation integrated models [20,21]. Benefiting from these important research findings, the majority of schools around the world now have implemented prevention and control efforts that are based on safety education for school violence, with proactive interventions such as increased surveillance equipment and patrols [22]. Different countries have also developed specific and effective experiences, such as Ireland [23], the United States [24], and South Korea [25], which have incorporated school violence into their youth legislation and policies to regulate it. Specifically in Finland, the Ministry of National Education has initiated the development of a curriculum system plan for the protection of students against violence in schools [26]. The Ministry of Education of Korea has launched the Regional Professional Programme to guide the prevention and follow-up of school violence through the development and implementation of special local programs that are led by schools and communities [27]. In addition, the Olweus Bullying Prevention Programme at Clemson University, USA, including whole-school, classroom, individual, and community components, was designed and evaluated for elementary, middle, junior high, and high school (K-12) and has been validated in relevant evaluation studies [28]. These social interventions are ideal for indoor violence prevention but are not so effective for outdoor violence. However, the lack of access and lighting in outdoor school environments [29,30] greatly increases the likelihood of violence, making it equally important to conduct research on outdoor school environments to prevent outdoor violence. Newcastle University in the UK launched an experiment called “Watchful Eye” [22] in response to the frequent theft of bicycles on campus. By putting up posters to psychologically intimidate potential criminals, the number of bike thefts on campus was halved after 12 months. Sandy Hook Elementary School in the USA was the scene of a serious school shooting in 2012, in which a number of students and teachers were killed. In the aftermath, Jay Brotman redesigned the school in the context of crime prevention theories [31]. Using a sample of high schools in New Jersey, USA, Aggarwal J compared data to find a stratified relationship between schools (buildings and facilities) and potential gun violence harborage, noting the need for timely response mechanisms between schools and government [32]. Further, Ning Ma of the Beijing University of Technology, China, designed a safety assessment method for the visually impaired based on the Visual Access and Exposure model of the outdoor campus environment, providing guidance for design and optimization [33]. As can be seen, although there has been some empirical research on school violence prevention through environmental design, it is mostly qualitative research and practice. Therefore, the outdoor school violence distribution (OSVD) should be fully considered in relation to the outdoor campus environment (OCE) in order to increase the design effectiveness.

In response, a large number of scholars have carried out long-term research and practice, and a more complete theoretical system has been formed for the study of the relationship between criminal behavior and spatial environment [34,35,36,37]. In 1961, Jane Jacobs analyzed the security of street space from a sociological perspective [38] and introduced the concept of the “Street Eye”, while in 1971, C. Ray Jeffery first developed the first generation of Crime Prevention Through Environmental Design (CPTED) theory [39]. Then, the concept of “Defensible Space” was first put forward by Oscar Newman in 1972 [40], advocating for the use of small plots in residential planning to provide more opportunities for surveillance in order to create a community environment that is manageable for residents. This theory is widely used in housing, retail, commercial, transport nodes, schools, hospitals, town centers, and stadiums. In 1982, James Q. Wilson and George L. Kelling introduced the Broken Windows theory [41] which states that the environment in which there is a lack of maintenance management will give a positive signal to potential criminal behavior. After development over the years, the second generation of CPTED theory that was proposed by Greg Saville and Gerry Cleveland is no longer limited to physical environment design [41], but emphasizes the importance of community participation, forming a theory system with territoriality, natural surveillance, access control, activity support, image and maintenance, and target hardening as the main body. This theory aims to reduce crime opportunities through design means such as enhancing natural surveillance, establishing clear site attributes, and maintaining the image of the spatial environment, and has been widely used in space types such as urban residential areas [42,43,44], green parks [45,46,47,48], greenways [49], urban renewal [50,51,52], tourist attraction sites [53], hospitals [54,55], college towns [56], and public transportation [57]. But only North American countries, represented by the United States and Canada, as well as Korea, Australia, Turkey, and Japan have developed crime prevention guidelines based on it [37] and applied it to educational buildings such as campuses [36,58]. To date, research on the relationship between spatial environment and criminal behavior, as represented by CPTED theory, has never ceased. Liu He and Kwan-Seon Hong from Dongseo University in Korea have already proposed the idea of the third generation CPTED through Double Diamond [59], and numerous cutting-edge studies on violence prevention visual mapping [60] and environmental diagnosis [61] have emerged, with more innovative research in progress.

Since the 1990s, there has been a shift from qualitative to quantitative research, with an increased focus on urban spatial and geospatial activities, particularly the impact of factors such as urban spatial structure, land type, and community units on crime, and the use of geospatial techniques to aid in the investigations [62,63,64], but less attention has been paid to the outdoor campus environment. Among them, GIS was introduced in China as early as the 1970s and gradually became an important application technology for the Chinese public security industry around 2000, resulting in the Police Geographic Information System (PGIS) [65]. In 2008, the US National Institute of Justice also held several seminars on crime prevention, proposing to cross-analyze criminal behavior by combining geography and visualization techniques to support subsequent prevention research [66]. In addition, some scholars have constructed a framework model for identifying spatial crime hotspots in cities by collecting real crime datasets and visualizing with GIS technology, taking a city as a research sample [67]. Apart from GIS technology, some studies have also used Spatial Syntax theory to analyze the impact of spatial layout on crime in communities or cities [68,69], such as Noha Ahmed Abd El AZIZ, who used this method to measure the safety of small- and medium-sized urban parks [68]. The Spatial Syntax theory here is a quantitative analytical model that is used to describe the quality of urban built spaces, proposed by Bill Hillier and Julienne Hanson in the 1980s [70]. Based on the topological properties of space, the theory provides a range of tools to analyze the linguistic characteristics of space, such as integration, configuration, user choice, and visibility, thus becoming an important tool for analyzing the relationship between spatial environmental design and criminal behavior. A small number of studies on campus security have been presented, such as Sherman SA’s use of mobile geoinformation technology to explore the contribution that is made by university police in the outdoor environment security [71]. Regrettably, most of these studies above have focused on criminal behavior in urban public spaces, and some of the studies on school safety do not address purely the features of the OCE. Therefore, there has been an urgent need for quantitative research on school violence to fill this gap, based on previous research in these similar fields.

In summary, facing the growing problem of school violence, although there are already social means such as security patrols and safety education, as well as physical interventions such as CPTED theory being developed, it is crucial to conduct quantitative research on the relationship between the OSVD and OCE to achieve effective prevention, which is still a gap. Thus, to fill the above-mentioned research area, the aim of this study is to quantitatively investigate the relationship between the OSVD (including teacher-student conflict, verbal bullying, physical conflict, and external intrusion) and the spatial attributes of the OCE (including the Integration, Mean Depth and Connectivity of spatial configuration, as well as the Visibility Connectivity of spatial visibility) through a case study, based on questionnaires, GIS techniques, and Spatial Syntax theory with its software. Through correlation and multiple regression analysis, regression models between the distribution of each outdoor school violence type and the outdoor campus environment then were developed, indicating the corresponding spatial attribute predictors. The results may provide guidance and theoretical support for many schools in planning, designing, and renovating outdoor environments for safety.

The research questions addressed in this paper include:

(1) What are the spatial distribution characteristics of different outdoor school violence types?
(2) Is there a relationship between the distribution of each outdoor school violence type and the spatial attributes (spatial configuration and spatial visibility) of the OCE? What is the relationship?
(3) How do the various spatial attributes of the OCE affect the distribution of each outdoor school violence type? What are the predictors?

## 2. Methodology

### 2.1. Study Design

**Figure 1:**
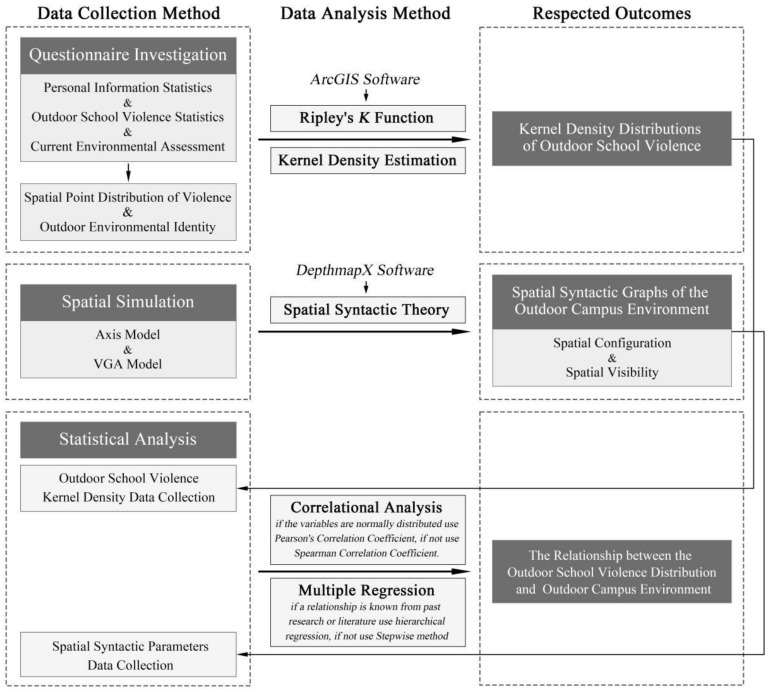
Schematic diagram of the research process framework.

Figure 1 displays the flow framework of this study, corresponding to the three sections of research questions, research methods, and the expected results. Firstly, the violence occurrence situation (types of violence, number of occurrences, spatial points of occurrence) and the current environment evaluation in the case school were collected through a questionnaire survey to produce a spatial point distribution map of outdoor school violence. Then the kernel density bandwidth values were calculated through Ripley’s K Function, and the kernel density values in the case school were carried out using the kernel density estimation to map the kernel density distribution of different outdoor school violence types. Secondly, based on Spatial Syntax theory, the spatial simulation analysis was conducted by building models of the OCE, and the vector distribution graphs of spatial configuration and spatial visibility were generated by using DepthmapX software. Finally, the statistical analysis, including correlation analysis and multiple regression analysis, was carried out to find out the causal relationship between various types of OSVD and the OCE, and to identify the regression models and predictors.

### 2.2. Field Site

This study was carried out in Case L, a boarding secondary school that is located in southern Zhejiang Province of China. The school was chosen as a case study for the following reasons.

- Universal school type: Statistics from Ministry of Education of the People’s Republic of China show that the number of boarding students in compulsory education has reached 32.765 million after 2012, accounting for 21.85% of the total number of students in school [72]. It is evident that boarding schools are common in China, and research on their school violence is somewhat applicable.
- Diversified building functions: In contrast to regular schools, boarding schools have facilities such as teaching buildings, office buildings, and gymnasiums, as well as dormitories and other facilities that serve the daily lives of students, which may increase the possibility of outdoor school violence to some extent.
- Special student population: Most of the students in boarding schools are left-behind children [73] whose parents are often in the laboring group. These students’ personalities tend to become stubborn, distrustful, lonely, and rude due to the lack of family care, as well as psychological problems such as anxiety, loneliness, and boredom that are caused by adaptation to the environment. Students in boarding schools are, therefore, a special group, with not only the general psychological characteristics of contemporary students, but also the special psychological problems of left-behind children.
- Representative violence rate: A random sample of students in different grades obtained an outdoor violence rate that reached 58.01%, which is higher than the above-mentioned violence rate in China (32.4%) and the global violence rate (32%), indicating that Case L is strikingly representative [68].

**Figure 2:**
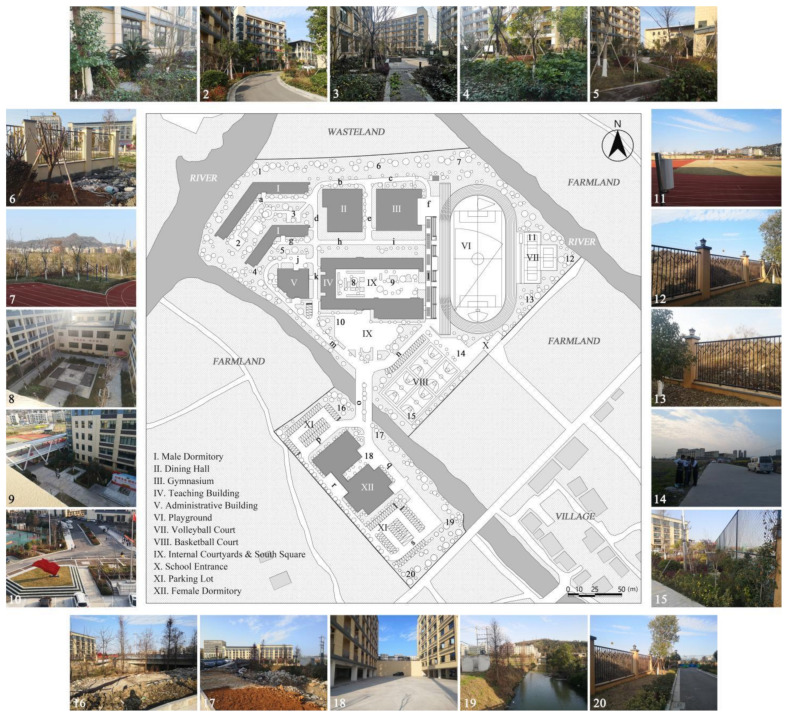
General plan of Case L with actual photos of main spatial nodes.

Consequently, Case L was finally selected for the study based on full consideration of the significance and generalizability of the findings. The students of Case L are mainly from the seventeen surrounding towns and eight neighborhoods, and are divided into three grades (freshman, sophomore, and senior) with a total of 27 classes and about 1000 students (10 classes in the freshman year, 13 classes in the sophomore year, and 4 classes in the senior year. The total number of seniors was small because most students transferred or dropped out of further studies). Meanwhile, the school covers an area of 67,333.3 m^2^ with a building area of 57,000 m^2^. Figure 2 illustrates that the school is separated into two areas by a river, with the northern part containing the teaching buildings, gymnasium, dining hall, male dormitories, and playgrounds, and the southern part including the female dormitories and park lots. As a supplementary, the roads (denoted by lowercase letters a to s) and the main spatial nodes with actual photographs (denoted by Arabic numerals 1 to 20) all have been marked one by one. In addition, it can be noticed that the Case L has three playground areas, a square with a garden, and several landscape nodes. There is also currently only one exit from the school, located to the north-east of the basketball court, which connects to the road leading to the town.

### 2.3. Questionnaire Investigation

#### 2.3.1. Investigation Method

In cooperation with teachers and students, the research team used recess time to administer questionnaires to students from different grades by using a combination of stratified and random sampling. More specifically, the three grades (998 students in total) were divided into three tiers, i.e., the freshman year as a tier (376 students), the sophomore year as a tier (462 students), and the senior year as a tier (160 students). As suggested by William G. Cochran [74], a further random sample of approximately 35% tested students was then selected for each tier to participate in the survey, comprising of 132 freshmen (35.11%), 162 sophomores (35.06%), and 56 seniors (35.00%), for a total of 350 tested students (1:1 male to female ratio), so as to achieve a certain representativeness, as detailed in Table A1. As a complement, the inclusion criteria for the sample were regular students that were enrolled in Case L who were informed and consented to participate in this study, while the exclusion criteria were students with mental disorders, cognitive impairment, or who were unable to cooperate for other reasons. Table A2 shows the details of the questionnaire, which consisted of three sections: personal information statistics (non-scale questions), outdoor school violence statistics (non-scale questions), and current environmental assessment (scale questions). The first part was designed to find out whether the composition of the tested students was balanced and reasonable. The second part aimed to understand the types of violence, the number of occurrences, and the spatial points of occurrence through item quizzes and spatial point markers. The third part was intended to support the subsequent exploration of the relationship between the OSVD and OCE by means of the subjects’ satisfaction with the current outdoor environment (16 questions were set in conjunction with the literature [75], containing 5 scales).

Once the questionnaires were returned, individual data samples needed to be screened and processed to improve the accuracy and completeness of the underlying data based on the following criteria [76,77].

- Deletion: When faced with invalid responses throughout the questionnaire, the sample should be simply deleted.
- Partial deletion: When an individual question in a valid questionnaire had a missing response, the data for it were removed but the other valid variables were retained.
- Modify: When faced with a missing variable in a sample, it can be filled in with the mean of the remaining variables. For classification, when the missing amount was less than 5%, the overall mean of the variable was used to replace. Otherwise, the hot deck method can be used to group the valid data for that variable and choose the mean of a certain group as the value of the missing sample.

Finally, a total of 342 questionnaires were returned, with a return rate of 97.71%. After screening and collating, 338 valid questionnaires were counted, resulting in the effective rate of 96.57%. SPSS19.0 was then used to complete the data entry and obtain the sample basic data after statistical analysis.

#### 2.3.2. Investigation Findings

The part “Personal Information Statistics” showed that among the 338 students participating in the survey, 167 were male (49.41%) and 171 were female (50.59%). According to the source of students, the largest number of students came from rural areas, reaching 291 (86.09%), while the smallest number of students came from suburban areas, only 17 (5.03%), and 30 (8.88%) from urban areas. And by the grade, 127, 160, and 51 students participated for freshman, sophomore, and senior, respectively, accounting for 37.57%, 47.34%, and 15.09%, correspondingly. Additionally, according to whether they were left-behind children or not, there were 87 left-behind students and 252 non-left-behind students, accounting for 25.74% and 74.56%, respectively.

**Figure 3:**
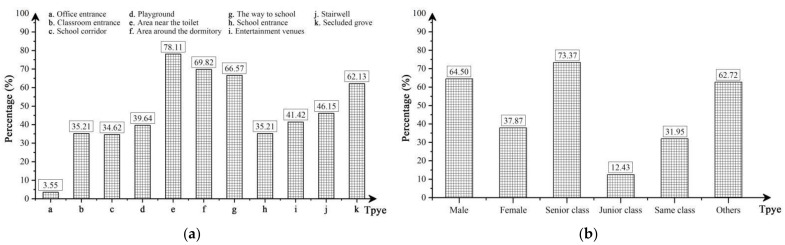
(**a**) Outdoor violence location; (**b**) Perpetrator type. *N* = 338, 50.59% female.

The part “Outdoor School Violence Statistics” indicated that more than 50% of the students reported having participated in, experienced, or observed different types of school violence, and the average incidence of outdoor violence for Case L was calculated to be 58.01%, as detailed in Table A3. The fact that Case L had such a high violence incidence, on the one hand, was inevitably linked to the nature of boarding school it is (diversified building functions, special student population, etc.), which also confirmed by numerous local studies in China [78,79,80]. On the other hand, the statistics included violence incidents that were experienced by perpetrators, victims, and bystanders, which may have been duplicated, resulting in some error. Overall, the school violence incidence in Case L still remained high and representative. In particular, as presented in Figure 3, the area near the toilet (78.11%), the area around the dormitory (69.82%), the way to school (66.57%), and the secluded grove (62.13%) were identified as the places where the most outdoor violence occurred. Males (64.50%) and seniors (73.37%) were considered to be the most dominant perpetrators, and 12.13% of the tested students indicated the presence of teacher violence. Furthermore, with the previous relevant references [81,82], these violence incidents were grouped into four categories, namely teacher-student conflict, verbal bullying, physical conflict, and external intrusion. Table 1 lists the number of occurrences and the spatial distribution maps of the four types of outdoor violence by coding and statistical processing (marked by the tested student). Among them, teacher-student conflict and verbal bullying were the most common violence behavior (the combined numbers of perpetrators and victims were 185 and 426, respectively), although physical conflict and external intrusion were not as prevalent (the combined numbers of perpetrators and victims were 130 and 76, respectively), it was also a strong sign of the seriousness for outdoor school violence in Case L.

The part “Current Environmental Assessment” was the scale part, so its reliability and validity were tested by using IBM SPSS Statistics 26, proving that the findings were reliable, with the detailed analysis process in Appendix B. The results showed that more than 20.00% of the tested students felt that the entrance to the school was narrow and disorderly (29.00%) and that the outdoor sports fields, for example, were far from the dormitory area (20.12%). In addition, more than 17.00% of the tested students believed that the current outdoor environment still has problems such as dead space in the landscape (19.23%), dim lighting design (17.16%), remote location of the dormitory building (18.34%), lack of leisure facilities (17.46%), more visual blind spots (18.94%), and so on. On the contrary, only around 6.00% of the tested students disagreed with the “poorly managed green space” and “untimely litter removal”. Complementary, the students’ agreement ratings with issues such as “low campus fence” and “confusing planning of school buildings” can be found in Table A4.

#### 2.3.3. Data Handling Tools

After obtaining the spatial point distribution maps for the four outdoor violence types in Case L, further speculative analysis was needed to get the density of violence distribution across the outdoor environment. As a result, Ripley’s K Function and kernel density estimation were applied in ArcGIS software. Firstly, Ripley’s K Function was used for pre-processing the spatial point distribution of outdoor violence occurrence to determine the distance parameter (kernel density bandwidth value) for kernel density estimation [83]. The formula is shown in Equation (1). After calculating Ripley’s K expected and observed values, the best clustering distances for teacher-student conflict, verbal bullying, physical conflict and external intrusion were determined to be 33.04 m, 70.50 m, 52.38 m, and 32.52 m, respectively.

(1) $$L\;(d)=\sqrt{\frac{A\sum_{i=1}^{n}\sum_{j=1,j\neq i}^{n}k_{i,j}}{\pi \cdot n\;(n-1)}}$$

*d* is the spatial radius scale, *n* is the total number of elements in the study area, *A* is the total area of the study site, and *k* is the number of elements in the set.

Secondly, with the advantages of intuitive representation, conceptual simplicity and ease of computer implementation, kernel density estimation was used to estimate the distribution of violence occurrence density, forming a two-dimensional smoothed estimation surface to reflect the characteristics and spatial variation of outdoor violence clustering in Case L. The specific formula is as Equation (2) [83]. x*_i_* is the point where the highest density of violence occurs. The further outward distance x*_i_* is the corresponding density value will decrease, and when the distance reaches a certain threshold, its density value will be close to zero. Ultimately, the kernel density distribution for each type of outdoor school violence was calculated by the above two steps.

(2) $$f(x)=\sum_{i=1}^{n}\frac{1}{h^{2}}\cdot \lambda \cdot (\frac{x-x_{i}}{h})$$

*f(x)* is the kernel density at point *x*, *n* is the number of points whose distance from *x* is equal to or less than *h*, *h* is the distance decay threshold (kernel density bandwidth value), and *λ* is the spatial weight function.

### 2.4. Spatial Simulation

DepthmapX, a tool of Spatial Syntax theory, was used to analyze the OCE in Case L. Specifically, it is a software is dedicated to urban spatial analysis and contains three basic models, namely Axis model, Convex Space Analysis (CSA) model, and Visibility Graph Analysis (VGA) model [71], which can be used to calculate parameters such as integration, choice and visibility. The Axis model and VGA models were applied in this study to analyze the relevant parameters of the spatial configuration and spatial visibility of the OCE, mainly described as following.

- Integration (In) represents the relationship between a space and local or overall space, that is, the accessibility of the space. The higher the In value, the higher the accessibility [73].
- Mean Depth (MD) indicates the number of transformations from local space to other parts of space, representing the convenience of the node in the spatial system. Higher values of MD indicate higher spatial separation [84].
- Connectivity (Con) refers to the sum of the number of spaces directly connected with the surrounding space. The higher the Con value of a space, the more spaces connected with it, characterizing as a transportation hub in the spatial system.
- Visibility Connectivity (VC) shows the number of other points that a point can see within its line of sight, reflecting the quality of natural surveillance provided by the outdoor environment to users or passers-by. The higher the VC value, the better the quality of surveillance or under surveillance [68].

These parameters that are mentioned above provide a comprehensive characterization of the accessibility and visibility capabilities of the OCE. The specific range of values in the final analysis for each parameter is expressed in a color scale from dark blue to dark red, with the former indicating low values and the latter indicating high values.

It is important to note that elements such as vegetation and street furniture within the environment need to be subdivided before conducting spatial simulations, as their impact on spatial configuration and spatial accessibility analysis is complex [46]. On the one hand, the height of the canopy bifurcation affects the spatial analysis, e.g., trees with a high bifurcation do not affect sight and behavior, but trees (especially shrubs) and street furniture that are too short can impede crossing. On the other hand, heavy foliage and bulky street furniture can obscure sight, while sparse foliage and furniture (such as utility poles) can provide some visibility. Therefore, a survey of the components of the outdoor environment in Case L was carried out to classify how they were calculated [68], as shown in Table 2. To summarize, first, shrubs and small trees with large crowns and low forks are defined as a hindrance. Second, large trees with extremely high forked canopies or small canopies, as well as low shrubs, resting seats, street lights, and other street furniture, are considered to be negligible obstructive factors. Third, motor vehicle parking is regarded as a hindering factor. In addition, considering the influence of tree canopy changes in different seasons on the spatial analysis, two seasons, summer (VC_S_) and winter (VC_W_), were chosen for this study to obtain more accurate and comprehensive results.

### 2.5. Statistical Analysis

After the above processing, the results of the kernel density of the OSVD and the spatial attribute variables of the OCE were derived. Statistical analysis then was conducted to explore the correlations and regression relationships between them [85]. As illustrated in Figure 2, roads and major spatial nodes in the OCE have been labeled to link their outdoor violence kernel density to the spatial attributes. With interpolation, the kernel density values of the various types of outdoor violence and the values of the four spatial attributes derived from DepthmapX (In, MD, Con, VC_M_ averaged over VC_S_ and VC_W_) were embedded in the same folder, via IBM SPSS Statistics 26 and Stata 15.1 software. Then they would be analyzed by correlational analysis and multiple regression, including the following specific steps.

- Normal distribution test: It was required for all data to select the appropriate correlation coefficient through this test. After embedded in SPSS Statistics 26, the one-sample Kolmogorov–Smirnov (K-S) test thus must be performed. If the variables were normally distributed (*p* > 0.05), Pearson’s correlation coefficient would be used, otherwise (*p* ≤ 0.05), Spearman correlation coefficient should be used [86].
- Correlation analysis: With the data imported into Stata 15.1 software, the bivariate correlation analysis was carried out using the appropriate correlation coefficients for kernel density variables and spatial attribute variables, discriminated by significance *p* and correlation coefficient r [85]. *p* was used to test whether there was a statistically significant relationship between the two variables (*p*-values less than 0.1, 0.05, or 0.01 all indicate a correlation, denoted by *, ** and ***, respectively, in ascending order of significance). If significant, the positive and negative direction of the correlation coefficient r and the degree of correlation should be analyzed. A larger absolute value of r indicated a higher degree of correlation.
- Multiple regression analysis and model building: It was used to explore the regression relationships between outdoor school violence kernel density variables and spatial attribute variables to predict the magnitude of the effect of different spatial attribute variables on the same violence kernel density. Specifically, the hierarchical regression method was used if the relationship between certain variables can be obtained from previous literature, otherwise the stepwise method should be chosen [85]. Once the regression analysis was determined, the data were tested for covariance to prevent interactions within the variables, including two indicators of variance inflation factor (VIF) (less than 10) and tolerance (greater than 0.2). Further, the regression coefficient beta, t-test value, and significance *p* were selected as indicators to discriminate the regression relationship. Among them, the t-test and *p*-value were judged by the same criteria as the above conditions. If the absolute value of beta was greater than zero, then the independent variable can effectively predict the variation of the dependent variable, i.e., there was a significant influence relationship. Also in the hierarchical regression analysis, R^2^ change (the difference in R^2^ between two consecutive models) represented the contribution that was made by increasing the independent variable; the larger the value, the more prominent the influence relationship.
- Regression model checking: The validity, reliability, and generalizability of the model all should be tested in this step. Combined with previous literature [87,88,89], the difference between R^2^ and adjusted R^2^ could indicate the generalizability if the value was closer to zero. The Durbin–Watson statistic needed to be in the range of 1.0–3.0, and the maximum value of Cook’s Distance for all samples should be controlled to be less than 1.0 to demonstrate that the regression model had good reliability. It was also necessary to determine whether the regression residuals were approximately normally distributed based on normal Q–Q plots, with a better fit indicating a closer to normal distribution. As a complement, the results of the correlation and regression analysis needed to be combined to define the effective predictors of the regression model.

## 3. Results

### 3.1. Kernel Density Distributions of Outdoor School Violence

**Figure 4:**
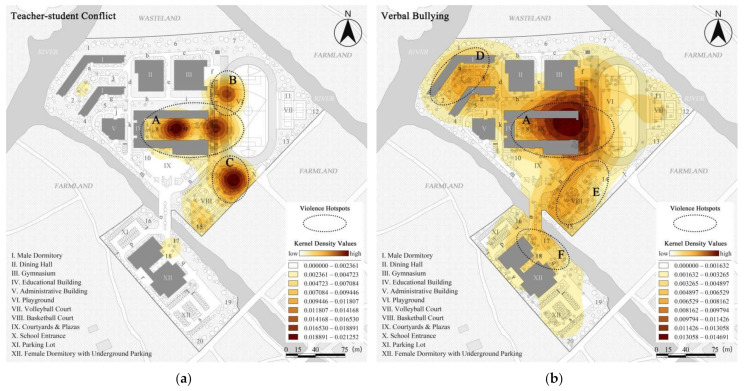

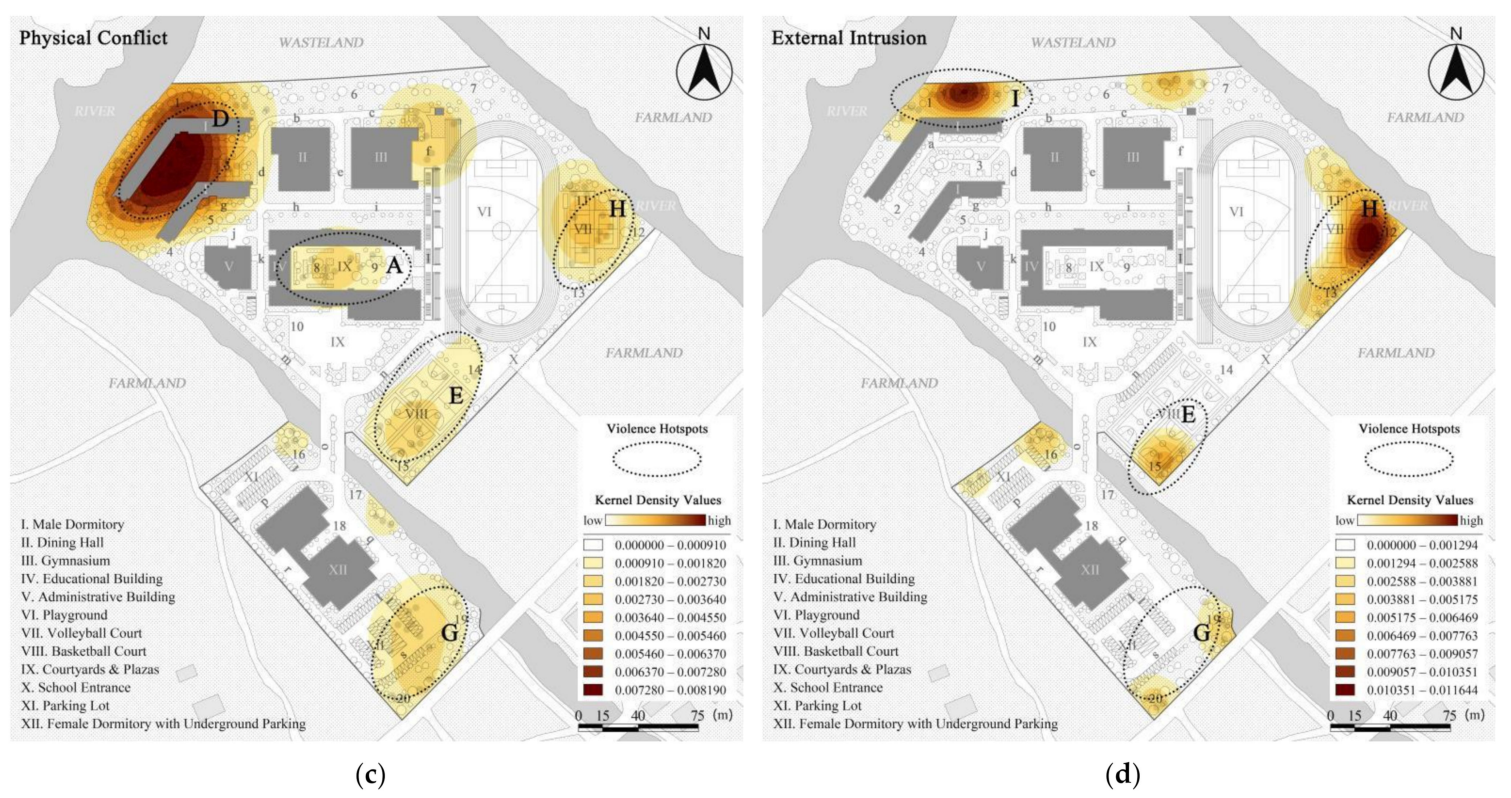
Kernel density distributions of various types of violence in Case L: (**a**) Teacher-student conflict (*N* = 185, d = 33.04 m); (**b**) Verbal bullying (*N* = 462, d = 70.50 m); (**c**) Physical conflict (*N* = 130, d = 52.38 m); (**d**) External intrusion (*N* = 76, d = 32.52 m).

Figure 4 illustrates the kernel density distribution of each outdoor school violence type formed from the questionnaire results, aided by ArcGIS 10.8 software. The aggregation degree of outdoor school violence is reflected by the distribution of kernel density values, i.e., the darker the color (brown), the higher the kernel density value and the higher the violence aggregation [83]. Firstly, Figure 4a shows the kernel density distribution of teacher-student conflict (*N* = 185, d = 33.04 m. The range of kernel density values is 0.000000–0.021252), the second most frequent type of violence, which can be seen to be concentrated mainly in the areas of the teaching building and school entrance. Therefore, areas A (internal courtyard), B (north-west of the playground), and C (entrance plaza) were marked as violence hotspots with corresponding maximum kernel density values of 0.02083, 0.01513, and 0.0212, respectively. Secondly, as indicated in Figure 4b, the kernel density distribution of verbal bullying (*N* = 462, d = 70.50 m. The range of kernel density values is 0.000000–0.014691), the violence type with the highest incidence has violence hotspots mainly in areas A, D (courtyard in the male dormitory), E (basketball court), and F (front plaza of the female dormitory), with a maximum and density of 0.01468, 0.00801, 0.00754, and 0.00548, respectively. Verbal bullying thus can be found to be widespread, with its presence in almost the entire outdoor environment and highly concentrated in two areas, the teaching building and male dormitory. Thirdly, the kernel density distribution of physical conflict (*N* = 130, d = 52.38 m. The range of kernel density values is 0.000000–0.008190) is shown in Figure 4c, with areas A, D, E, G (southern parking lot), and H (volleyball court) marked as violence hotspots with the highest kernel densities of 0.00223, 0.00819, 0.00230, 0.00247, and 0.00339, respectively. The physical conflict distribution was scattered, largely located in the corners of the OCE and highly clustered in the male dormitory area. Finally, Figure 4d represents the kernel density distribution of external intrusion (*N* = 76, d = 32.52 m. The range of kernel density values is 0.000000–0.011644), the type of violence with the lowest incidence. The results show that areas E, G, H, and I (the grove in the north part of the male dormitory area) were marked as violence hotspots, with maximum kernel densities of 0.00561, 0.00327, 0.01164, and 0.01041, correspondingly. As can be seen, while both external intrusion and physical conflict were mainly located in the corners, the former was only located in the campus boundary spaces (near the fence).

### 3.2. Spatial Syntactic Graphs of the Outdoor Campus Environment

Using DepthmapX, the boundary lines of walkable areas such as roads, squares, and outdoor sports fields were drawn to create an Axis model for the spatial configuration, i.e., the walkable layers. Meanwhile, the boundaries of the fence, the outer contours of the buildings, the contours of the plant canopy, and the boundaries of the different street furniture were mapped to establish a VGA model for the spatial visibility. In mapping the two models above, the lawn was defined to be a non-walkable layer and the parking lots were considered to be parked full of vehicles. Lastly, the analysis results were interpolated through ArcGIS to obtain specific values, as shown in Table 3.

#### 3.2.1. Spatial Configuration Graphs

**Figure 5:**
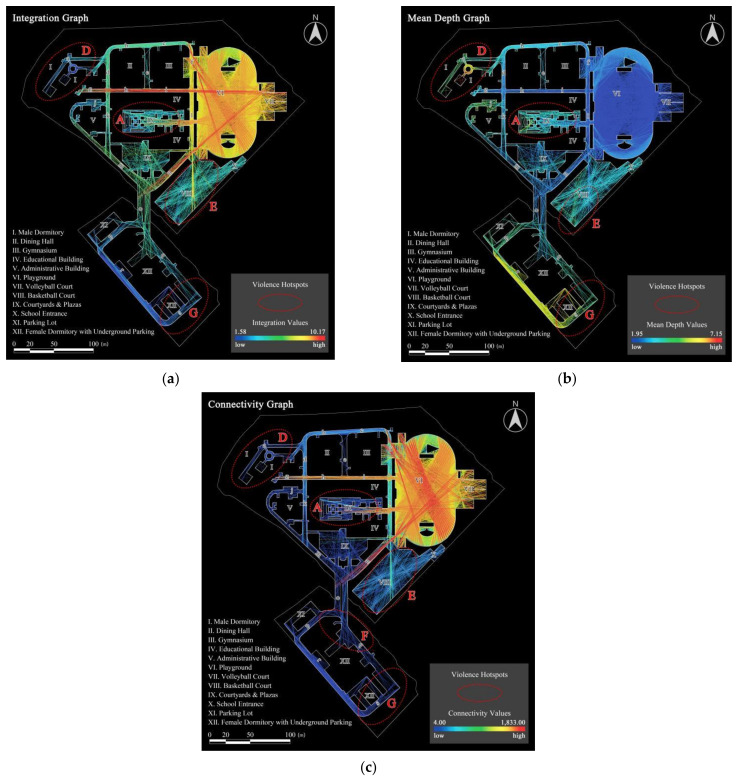
Spatial configuration graphs exported from DepthmapX software (areas A, D, E, F and G are the violence hotspots calculated through kernel density estimation in ArcGIS): (**a**) In graph; (**b**) MD graph; (**c**) Con graph.

Figure 5 indicates the simulation results of the spatial configuration of the OCE, including In, MD, and Con. The graphs show that the playground, volleyball court, and the roads connecting them (roads f, h, i, l, and n) were important areas in the OCE with the highest levels of In and Con (described in red) and the lowest MD (described in blue), which had the best accessibility due to the absence of obstacles and were identified as hotspots. In addition, several areas with moderate In, MD, and Con were found, including the basketball court, the south square, and the roads connecting them to the playground (roads b, c, d, k, m, and o), which were medium in accessibility (described in the neutral green). However, there are still a few traffic-clogged spaces that were regarded as less accessible spatial dead spots because they were at the end of the road network (road ends or narrow courtyards), such as the courtyards and roads around the male dormitory (roads a and j) and the roads near the female dormitory (roads q, r, s, and t), which got the least In and Con (described in blue) and the highest MD (described in red). In combination, the spatial configuration attributes in the OCE showed a clear polarization. Comparing with the kernel density distributions above, it can be found that outdoor violence mostly occurs in areas with lower In and Con, but higher MD, containing areas A, D, E, F, and G. It was supported by the fact that the most frequently agreed items in the “Current Environmental Assessment” of the questionnaire were “confusing campus building planning”, “remote location of the dormitory building”, “remote outdoor sports grounds”, “dead space in the landscape”, and “lack of leisure facilities”.

#### 3.2.2. Spatial Visibility Graphs

**Figure 6:**
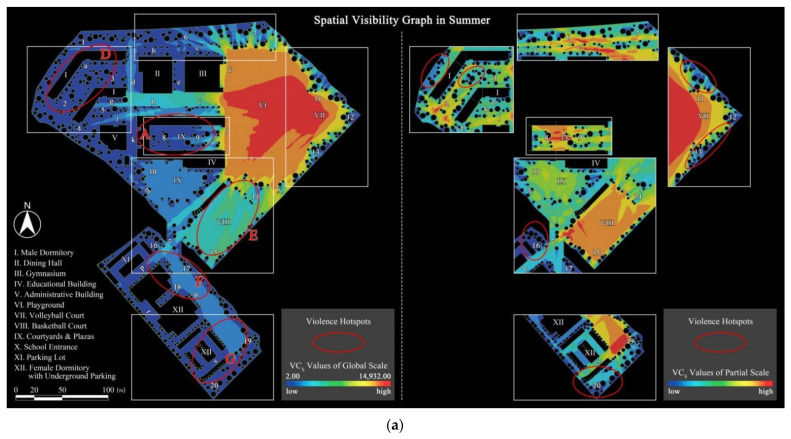

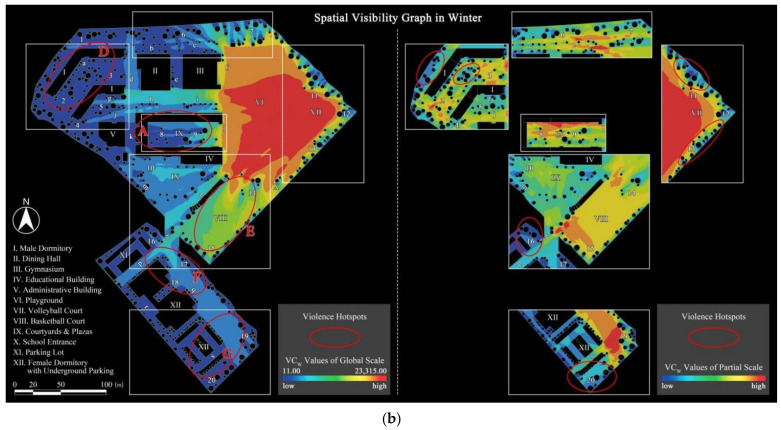
Spatial visibility graphs in two seasons (areas A, D, E, F and G are the violence hotspots calculated through kernel density estimation in ArcGIS): (**a**) Summer scenario; (**b**) Winter scenario.

The results of the spatial visibility simulation of the OCE of Case L are shown in Figure 6, containing both global and partial scales for summer and winter seasons. Globally, the visibility distribution remained strongly consistent in both two seasons. Specifically and firstly, the playground, volleyball court, and roads to the west of them (roads f and l) all showed good visibility (described in red) due to the openness of the space, especially the volleyball court, which was the area with the best visibility and is considered as a hotspot. The large number of trees planted along the outer boundary of the playground diminished the visibility of the area behind it, making the boundary area the least visible (described in blue). It means that the visibility of the outdoor sports field area was radially distributed, i.e., the central area was the most visible and the outer boundary areas were the least visible. Secondly, the spaces among the teaching building, the gymnasium, and the dining hall as well as the basketball court, were at an average level of visibility (described in the neutral green), with relatively average VC. Thirdly, the internal courtyard, the male dormitory area, and the parking lot around the female dormitory, which were shaded by the building recessed spaces and vegetation areas, presented a lower level of VC (described in blue) and were identified as the least visible spaces. Collectively, the spatial visibility in the OCE also showed clear differences, which, when compared to the outdoor violence kernel density hotspots, were found to be mostly concentrated in areas of poor visibility such as areas A, D, F, and G. Similarly, the “more visual blind spots”, which was highly endorsed by the tested students in the questionnaire, explained this phenomenon.

Furthermore, a comparative analysis of local visibility between the two seasons revealed that the spatial visibility was more negatively affected by deciduous trees, especially trees with medium-sized canopies such as *Liquidambar formosana* Hance, *Punica granatum* L., and *Prunus serrulata*. The effect was more pronounced in summer than in winter, as evidenced by the fact that the area of high visibility was significantly smaller in summer than in winter. Also, some of the spatial nodes (nodes 3, 4, 13, 14, 15, 19, and 20) were more visible in winter due to the seasonality of deciduous trees.

### 3.3. The Coupling Relationship between the OSVD and the OCE

Referring to the roads and main spatial nodes in the OCE of Case L, a square with a side length of 5 m was drawn at the center of 20 spatial nodes (comparing the total dimensions of Case L, 5 m is more appropriate) [90], and data on spatial attribute variables were crawled for four vertices and centroids, for a total of 100 sample sets. The kernel densities of the four outdoor school violence types were named KD1 (for teacher-student conflict), KD2 (for verbal bulling), KD3 (for physical conflict), and KD4 (for external intrusion). Importantly, the Axis model did not exactly overlap with the VGA model, as the former only included walkable space, while the latter covered roughly the entire outdoor environment. Therefore, the spatial configuration parameters (In, MD, and Con) of the non-viable layer were considered to be zero when interpolation was performed, but meanwhile the spatial visibility parameter remained valid and was replaced by the VC_M_ (average of VC_S_ and VC_W_). Then, the sample data were finally placed into IBM SPSS Statistics 26 and Stata 15.1 software for subsequent statistical analysis.

#### 3.3.1. Normal Distribution Test Results

To ensure the logical rigor, a normal distribution test was needed to be carried out to determine the appropriate correlation coefficient before statistical analysis. With IBM SPSS Statistics 26, the one-sample Kolmogorov–Smirnov test (K-S) was used and the results are shown in Table 4. The results indicated that the *p*-values of the K-S statistic for these variables were all less than 0.05, implying that all were non-normally distributed and the Spearman Correlation Coefficient was determined to be applied, as suggested by Andy Field [86]. Also, the magnitudes of some of the variables were adjusted, where statistically permissible, to eliminate the effects of large differences in numerical magnitudes between variables.

#### 3.3.2. Correlation Relationship

**Figure 7:**
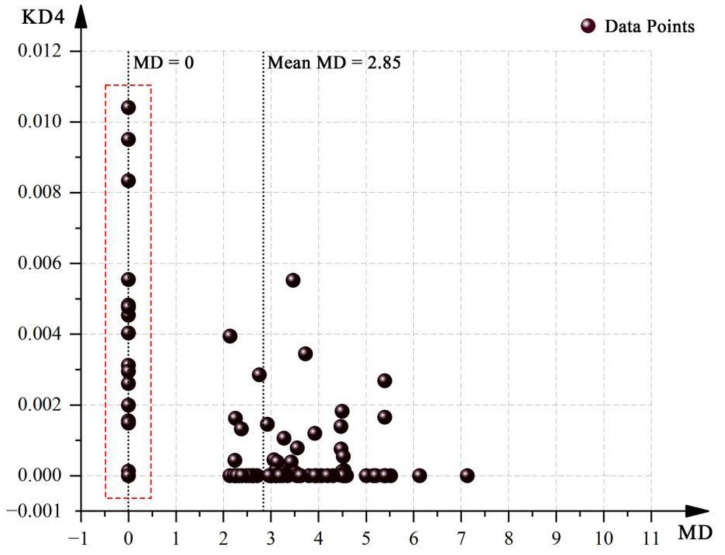
The relationship between KD4 and MD of Case L.

The correlation between the kernel densities for different OSVD maps and the spatial attributes of the OCE was assessed by applying the non-parametric Spearman Correlation Coefficient in Stata 15.1 software, and the results are shown in Table 5. KD1 was found to be related to all four spatial attribute variables, but all were moderately positively correlated (In: r = 0.246, *p* < 0.05; MD: r = 0.210, *p* < 0.05; Con: r = 0.200, *p* < 0.05; VC_M_: r = 0.242, (*p* < 0.05). Similarly, KD2 showed positive correlations with these spatial attribute variables (In: r = 0.332, *p* < 0.01; MD: r = 0.187, *p* < 0.1; Con: r = 0.290, *p* < 0.01; VC_M_: r = 0.293, *p* < 0.01), with In correlated the most strongly, while the MD had the weakest relationship. In contrast, KD3 presented a strong negative correlation with In in spatial configuration (r = 0.320, *p* < 0.01) and a moderate negative correlation with Con and VC_M_ (Con: r = 0.250, *p* < 0.05; VC_M_: r = 0.224, *p* < 0.05). At the same time, KD4 was identified to have a strong negative correlation with the MD (r = 0.392, *p* < 0.01), but no significant correlation with the other three variables. Figure 7 displays the relationship between KD4 and MD. Since some of the data points were collected from the non-viable layer, these points were distributed on axes with a MD value of zero, leading to the illusion of a negative correlation. In fact, most of the high kernel density points of external intrusion were concentrated in the non-walkable layer.

In summary, the results of Spearman correlation analysis indicated that as the values of the three spatial attribute variables of In, Con, and VC_M_ increased, both KD1 and KD2 in Case L grew, while KD3 decreased. Among these, verbal bullying was most significantly associated with all three, physical conflict was the next, and teacher-student conflict was the least relevant. Moreover, KD1 and KD2 were also related to MD and rose with its increase. KD4 correlated most strongly with MD and decreased with it increasing and increased most significantly when the MD value is zero (located in the non-viable layer).

#### 3.3.3. Regression Relationship and Models

To explore the nature of the relationship between the kernel density distributions and spatial attributes of each outdoor school violence that are mentioned above, and to complete the in-depth discussion from qualitative to quantitative analysis, a regression model for Case L was developed in Stata 15.1 software. Before the regression analysis, the four spatial attribute independent variables first needed to be analyzed for multiple covariance to determine whether there was some degree of co-linearity between them and to prevent any impact on the contributory nature of the model. Table 6 presents the results of the co-linearity analysis. All spatial attribute variables had VIF values less than 10 and Tolerance values above 0.2, indicating the absence of significant covariance, providing support for the validity of the regression model following, as suggested by Bruce L. Bowerman and Richard O’Connell [87,88].

Multiple regression analysis followed the approach pointed out by Field, A.P. and for this study, there has been no explicit quantitative research on the relationship between school violence and spatial attributes, so a stepwise method and standardized tests were conducted on all independent variables. For KD1, the values of R^2^ were boosted whenever an independent variable was added to the model, as displayed in Table 7. Among these, In and Con accounted for 5.7% and 7.0% of the R^2^ change in the teacher-student conflict kernel density. In the final model of Table 8, the standardized test revealed the effects of each predictor with two predictors, In and Con, found to be significant predictors of teacher-student conflict (In: beta = 10.334, t(100) =3.04, *p* < 0.01; Con: beta = 0.066, t(100) = 3.23, *p* < 0.01), with positive and negative effects, respectively. As the analysis progressed shown in Table 9, the R^2^ change for KD2 was relatively large at 11.2% and 1.9% when In and Con were added. In turn, the data from the final model in Table 10 verified the influence of In and Con in relation to verbal bullying (In: beta = 5.585, t(100) = 2.54, *p* < 0.05; Con: beta = 0.023, t(100) = 1.69, *p* < 0.1), with the former being a moderate positive effect and the latter a weak, negligible negative effect. For KD3 illustrated in Table 11, the value of R^2^ also increased significantly with each addition of the variable. The largest R^2^ changes were caused by In, MD, and Con, accounting for 8.4%, 4.9%, and 7.6%, respectively, in physical conflict kernel density accordingly. Meanwhile, as Table 12 exhibits, the standardized test also showed that these three independent variables significantly predicted the distribution of physical conflict with a negative effect of In and a positive effect of the other two variables (In: beta = 6.521, t(100) = 4.12, *p* < 0.01; MD: beta = 4.616, t(100) = 3.44, *p* < 0.01; Con: beta = 0.029, t(100) = 3.00, *p* < 0.01). The regression model for external intrusion was similar to that for physical conflict, as can be seen in Table 13 and Table 14, with In, MD, and Con also being significant predictors of the distribution, except that MD was associated with a moderate negative effect (beta = 3.510, t(100) = 2.42, *p* < 0.05).

#### 3.3.4. Regression Model Checking Results

**Figure 8:**
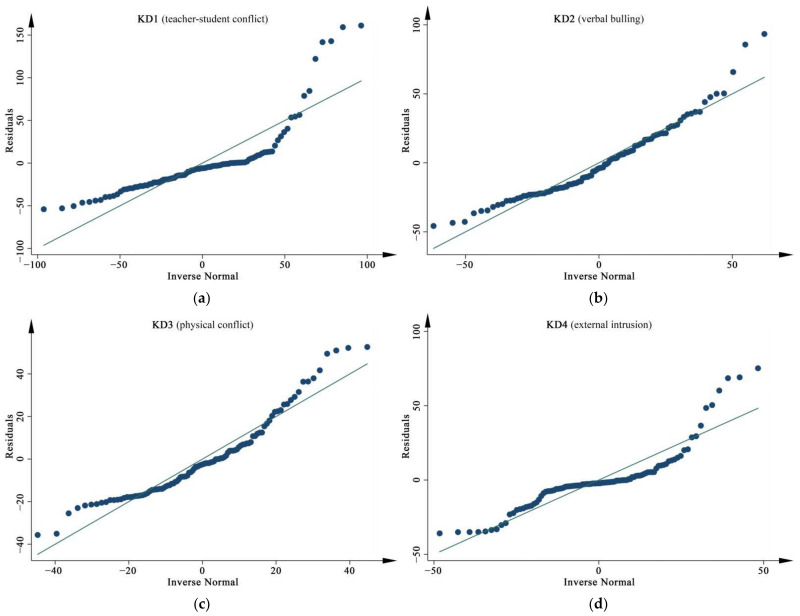
Normal Q-Q plots of the dependent variable, *N* = 100: (**a**) For KD1; (**b**) For KD2; (**c**) For KD3; (**d**) For KD4.

The model generalizability, the Durbin–Watson statistic, case diagnosis, and normality tests were carried out again to examine the validity of the regression models that were built above, as detailed in Table 15. Firstly, the difference comparison between the R^2^ and the adjusted R^2^ for the four final models shows that each model had a difference of around 0.03, i.e., occupied only a relatively small variance of around 3.0%. If the model was artificially driven conditions, this would suggest that all four models have good generalizability. Secondly, all values of the Durbin–Watson statistic were between 1.0 and 3.0, indicating that the assumptions under the dependence error were feasible, as Field [49] argued. Thirdly, the maximum values of Cook’s Distance for all the models were less than 1.0, presenting that the influence of a single sample on the overall regression model was within a manageable range. Finally, as can be seen in Figure 8, the probabilities for each of the regression models were normally distributed. The stability, validity, and generalizability of the models were supported by all the regression statistics that were analyzed.

Notably, the results of the regression analysis supported some of the conclusions of the correlation analysis, while some of the ideas were not confirmed. With reference to the relevant literature [89,91], correlation analysis is a bivariate relationship, while regression analysis considers the interaction between multiple variables, meaning that correlation is not necessarily related to the existence of a regression relationship. When there is a correlation between the variables, but no regression relationship, it is considered that only a correlation exists. And when there is no correlation but a regression relationship exists, no correlation will be accepted as a conclusion. However, when there is a negative (positive) effect but a positive (negative) correlation, there is concluded to be a correlation but no regression relationship. Consequently and lastly, in this study, In was found to have a positive effect with teacher-student conflict and verbal bullying, and a negative effect with physical conflict, but a relatively weak relationship with verbal bullying. In other words, In can be identified as a significant predictor of teacher-student conflict and physical conflict, and a general predictor of verbal bullying. MD were discovered to have a positive and a negative effect relationship with physical conflict and external intrusion, respectively, which, in principle, could be used as significant predictors of these two violence types. However, according to Figure 7, external intrusion usually occurred in spaces with a MD value of zero; therefore, it was not recommended as a predictor.

## 4. Discussion

Research on the relationship between criminal behavior and environmental space has been going on for about 60 years. Through the investigation and practice of numerous scholars, important theories such as the first and second generations of CPTED have been established and made significant contributions to safety construction in many aspects of urban settlements, streets, parks, and schools. In recent years, with the information age arriving, research in this field has gradually been linked to quantitative research methods such as geospatial technology, with ArcGIS and Spatial Syntax theory having become commonly used tools. However, over the years, most of these quantitative studies have focused on applications at urban scales but consistently paid little attention to school violence. To fill this gap, this study was purposed to explore the intrinsic relationship between the OSVD and the OCE, taking China, which has a high proportion of boarding schools, as the research context, and selecting Case L in southeastern Zhejiang province as the empirical case. By organizing the meticulous field survey and using ArcGIS and DepthmapX software, the kernel density distributions of different types of outdoor school violence and the spatial syntactic graphs of the outdoor campus environment were mapped. Through statistical analysis, the quantitative relationships between the four kernel density variables of outdoor school violence (teacher-student conflict, verbal bullying, physical conflict, external intrusion) and the four spatial attribute variables (In, MD, Con, VC_M_) were revealed, with their regression models developed and the corresponding predictors indicated, providing guidance for improving the safety of the outdoor environment.

Firstly, four important and major school violence types and their distributional characteristics were identified for Case L. Teacher-student conflict occupied the second highest percentage (22.64%), which was strongly related to the nature of boarding schools. Many studies have also shown that boarding schools were often characterized by functional and mixed buildings, specific physical and psychological problems of the student groups (rebellion, sensitivity, irritability, lack of family care), inappropriate management by teachers (full-day management of both study and life, and unreasonable teacher-student ratios), which led to numerous teacher-student conflicts such as corporal punishment. In addition, this kind of violence was mainly clustered within the area around the teaching building (the maximum kernel density was 0.021200), which was closely associated with the range of teachers’ activities [92,93]. Verbal bullying was the most prevalent of all the violence types (52.14%), both in terms of numbers and distribution characteristics, as it was a less difficult and more insidious form to perpetrate, as found by Wang Peng [78]. As a result, it was present in almost the entire outdoor environment and was concentrated in the teaching building and the dormitory area (the maximum kernel density was 0.01468). Physical conflict, with the third highest incidence (15.92%), was relatively costly to implement and needed to be carried out in spaces that were well concealed or easy to escape from, as summarized by Wang Juan [79]. Its distribution, therefore, was more dispersed and mostly located in the end of roads, courtyards, and other corners with less In and greater MD, such as the male dormitory area (the maximum kernel density was 0.00819), which was consistent with previous studies [94]. External intrusion, the violence type with the lowest incidence (9.30%), tended to occur in the border areas of the non-walkable layer, which was related to the activity characteristics of outsiders as they needed to seek the fastest escape routes [79,80,94].

Secondly, the correlation analysis results indicated that there was a relationship between the OSVD and all spatial attributes of the OCE, which corresponded to the findings of Kweon Jihoon, Ju Mi-Ok, and Lee Chang-Hun [95,96,97,98,99]. Specifically, teacher-student conflict, verbal bullying, and physical conflict were all found to be correlated with In, Con, and VC_M_ (for KD1, In: *p* < 0.05, Con: *p* < 0.05, VC_M_: *p* < 0.05. for KD2, In: *p* < 0.01, Con: *p* < 0.01, VC_M_: *p* < 0.01. for KD3, In: *p* < 0.01, Con: *p* < 0.05, VC_M_: *p* < 0.05.), with verbal bullying being the most significantly related. There are two main reasons why it was the most significant. For one, it had the largest sample size (*N* = 462), making it a more accurate calculation of kernel density values and possibly further highlighting the significance [91]. Another reason is that the distribution of teacher-student conflict was related to the range of teacher activities, with physical conflict also often occurring in areas of high In and low MD, such as outdoor sports fields, while the distribution of verbal bullying was relatively consistent, mostly in areas of high In and low MD [97,99]. The high coherence of the data thus strengthened the significance of the correlation between the variables. Furthermore, MD was also a spatial attribute that was associated with the presence of teacher-student conflict, verbal bullying, and external intrusion (KD1: *p* < 0.05. KD2: *p* < 0.1. KD4: *p* < 0.01). Within these, the most significant correlation between external intrusion and MD was due to the large number of spatial points that were distributed in the non-walkable layer (MD = 0), creating a prominent correlation [78,94].

Thirdly, the regression analysis results supported some of these findings and showed that In was a significant predictor of teacher-student conflict and physical conflict, and a general predictor of verbal bullying, while MD was a significant predictor of physical conflict, but not recommended as a predictor of external intrusion. As an extrapolation, it can be argued that the outdoor environment with lower In and higher MD may be the place where physical conflict is frequent, as verified in the spatial distribution maps in Case L and in line with the points made by Yijuan Qiao and her team [100,101]. For this violence space, it is possible to attract students and teachers to carry out communication activities and enhance territoriality by re-planning the road network or implanting crowd activities, such as landscaping vignettes, resting seats, and organizing outdoor activities [58,102]. Meanwhile, the environmental image should be well maintained. When choosing tree species, shrubs are mainly below the average height of students’ sight lines, while trees are mainly those with high forked crowns and small crowns. Teacher-student conflict tends to occur in areas with high In and low MD, and in the light of the relevant literature [78,79,103], it can be speculated that this may be related to the teachers’ activity phenomenon that tends to revolve around spaces with good accessibility, such as teaching buildings. In addition to social interventions such as ICC-T [104], the campus environmental design needs to strengthen the monitoring capacity, and activity inclusion of teachers to reduce the violence occurrence. For example, teacher management stations can be set up in areas of frequent student-teacher activity, including sports fields and courtyards, to reduce the violence incidence such as corporal punishment as a result of student rebellion [94]. The effects between verbal bullying and each spatial attribute are not significant enough, which may be related to the lower cost of violence occurrence, as Ku Na Hyoen and Wahab AA [105,106] argued. For prevention, electronic surveillance and safety information boards in crowded spaces will be important to restrain the behavior and motivation of potential perpetrators. For spaces with high In and low MD, in order to control the people flow and enhance the dwellability of the space, spatial variations can be enriched by adding flower beds, ponds, and other landscape features, appropriately increasing the MD and reasonably installing facilities to enhance surveillance and management [94,107]. In contrast, this study does not recommend MD as a predictor for external invasion, despite its significance, as external invasion tends to occur in the non-walkable layer of the boundary where the MD value is zero. For these spaces, on the one hand, higher or additional walls are needed to improve access control, and on the other hand, landscape design and image maintenance should be enhanced to reduce view shading by regularly building foliage or planting trees with high forked canopies to make it impossible for outside perpetrators to invade and hide [68,78,107]. In addition, to compensate for the poor visibility of spaces, increased lighting and electronic surveillance equipment are also necessary to improve the public safety of the outdoor campus environment. The quantitative findings and environmental security optimization tools that are summarized above are all interpreted accordingly in the qualitative analysis of the relevant literature, further validating the reliability of the predictors.

Finally, there are still numerous limitations and shortcomings in this study. Above all, only one boarding school was selected as a sample, which has the restriction of a small sample and is not representative of the specific situation of school violence across China or the world as a whole, and more further case studies are needed to validate this. Also, the outdoor violence distribution maps were plotted by marking the violence locations on the general plan, which was not a fully accurate method of counting and relied on student memory, with data subject to error. Nevertheless, it could still be a positive help in determining the general areas where outdoor violence occurs. It would be a more reliable approach to use incidents of violence that are registered with the school security office as a source of data. Moreover, many difficulties were identified in the simulations that were carried out using DepthmapX. On the one hand, the outdoor environment does not have clear physical boundaries, such as walls, so the spatial configuration analysis classified the lawn as an inactive area, even though people can step on it. On the other hand, as the software is a two-dimensional operating environment, it does not yet support the analysis of influencing factors in three-dimensional space. Although this study categorized possible obstacles in the outdoor campus environment to get as close to the real results as possible, it ignored the influence of factors such as road materials and environmental color [108,109], which still needs to be improved with the help of 3D simulation software and a more thoughtful evaluation system.

## 5. Conclusions

Despite the obvious limitations of the small sample and representativeness, this study makes a contribution to research on school violence prevention through environmental design. At the same time, nowadays, there are few studies that use quantitative techniques to explore the distributional characteristics of school violence, where the innovation of this study lies. Through questionnaire investigation, ArcGIS, and Spatial Syntax theory, the relationship between the OSVD and the OCE was explored and the corresponding predictions were determined, highlighting and validating the intrinsic role of spatial attributes of the OCE (spatial configuration and spatial visibility) on the of OSVD. Specifically, In was noted as a significant predictor of teacher-student conflict and physical conflict (KD1: beta = 10.334, *p* < 0.01; KD3: beta = 6.521, *p* < 0.01), a general predictor of verbal bullying (beta = 5.585, *p* < 0.05), while MD was found to be a significant predictor of physical conflict (beta = 4.616, *p* < 0.01). Therefore, by calculating the In and MD of the spatial configuration, combined with VC_M_, a significantly relevant variable, it is possible to predict more accurately the potential places where school violence will occur. And then environmental design strategies can be carried out in a targeted manner, such as enhanced surveillance, activity support, and image maintenance for different spatial attributes, thereby increasing effective prevention. Such a predictive model would allow the establishment of a guideline for the security campus planning, as well as setting up an evaluation mechanism to predict and test design solutions, which would have great potential and practicality to provide guidance and evidence for the outdoor environments design to prevent school violence. It is a brand-new attempt, but in order to get a more accurate and reasonable prediction method, the field needs to be improved with more types and numbers of school samples, more accurate and objective violence data, and more comprehensive software for 3D spatial simulation (the quantitative calculation of environmental color and texture), etc.

## Figures and Tables

**Table 1:** The number of occurrences and the spatial point distribution maps for the four outdoor violence types in Case L.

Violence Types	Violence Activities	Number	Total Percentage (%)	Spatial Distribution Map
Teacher-student conflict	Cynicism	93	22.64	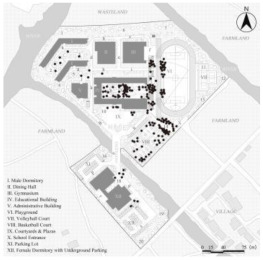
	Corporal punishment	92		
Verbal bullying	Insulting nickname	98	52.14	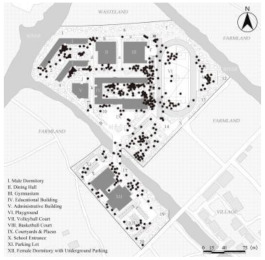
	Isolating others	90		
	Ridiculing others for the incompatibility of their personality with their gender	44		
	Deliberately pushing others	42		
	Verbal threat	64		
	Refusing others to participate in collective activities	39		
	Reject people of the same gender or opposite gender	49		
Physical conflict	Kicking and beating	89	15.92	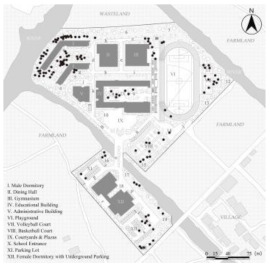
	Fighting	41		
External intrusion	Intimidation and threat	36	9.30	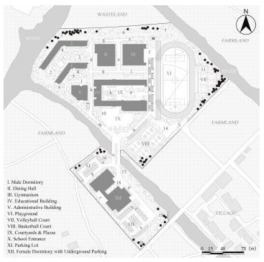
	Blackmailing	29		
	Affray	11		

Note: These statistics of violent incidents were obtained by marking on a general plan.

**Table 2:** Calculation of the elements of the OCE in the spatial simulation.

Object	Type	Trunk Height	Canopy Size	Opacity in Summer	Opacity in Winter
*Cinnamomum camphora*	Evergreen tree	Medium	Large	High	High
*Ligustrum lucidum*	Evergreen tree	High	Large	Low	Low
*Ginkgo biloba*	Deciduous tree	Medium	Small	High	Low
*Metasequoia*	Deciduous tree	Medium	Small	High	Low
*Osmanthus fragrans*	Evergreen tree	Medium	Medium	High	High
*Magnolia grandiflora*	Evergreen tree	High	Large	Low	Low
*Camellia japonica* L.	Evergreen tree	Low	Medium	High	High
*Liquidambar formosana Hance*	Deciduous tree	Low	Medium	High	Low
*Liriodendron chinense*	Deciduous tree	High	Large	Low	Low
*Punica granatum* L.	Deciduous tree	Low	Medium	High	Low
*Prunus serrulata*	Deciduous tree	Low	Medium	High	Low
*Sapindus*	Deciduous tree	High	Large	Low	Low
Basketball hoop	Sporting facility	/	/	Low	Low
Football rack	Sporting facility	/	/	Low	Low
Street light	Infrastructure	/	/	Low	Low
Rubbish bin	Infrastructure	/	/	Low	Low
Outdoor seat	Infrastructure	/	/	Low	Low
Parked car	Infrastructure	/	/	High	High

**Table 3:** Descriptive statistics of the spatial attribute values for the Case L.

Spatial Attribute	In	MD	Con	VC_S_	VC_W_
Mean	6.10	2.85	687.29	13,818.00	16,470.00
Minimum	1.58	1.95	4.00	2.00	11.00
Maximum	10.17	7.15	1833.00	14,932.00	23,315.00
Std. deviation	1.98	0.88	574.55	13580.09	15125.83

**Table 4:** Test results of normality.

Indicators	KD1	KD2	KD3	KD4
K-S statistic	0.295	0.321	0.190	0.307
Sig.	0.000 ^a^	0.000 ^a^	0.000 ^a^	0.000 ^a^
**Indicators**	**In**	**MD**	**Con**	**VC_M_**
K-S statistic	0.109	0.137	0.352	0.251
Sig.	0.004 ^a^	0.000 ^a^	0.000 ^a^	0.000 ^a^

^a^ Significant values after Riley’s correction. Note: Significant values for the K-S test indicate deviations from normality.

**Table 5:** The relationship between the OSVD and the OCE in Case L.

Variables		KD1	KD2	KD3	KD4
In	Spearman Coefficient	0.246 **	0.332 ***	−0.320 ***	−0.152
	Sig. (2-tailed)	0.012	0.001	0.001	0.121
MD	Spearman Coefficient	0.210 **	0.187 *	0.075	−0.392 ***
	Sig. (2-tailed)	0.032	0.057	0.448	0.000
Con	Spearman Coefficient	0.200 **	0.290 ***	−0.250 **	−0.094
	Sig. (2-tailed)	0.041	0.003	0.010	0.338
VC_M_	Spearman Coefficient	0.242 **	0.293 ***	−0.224 **	0.028
	Sig. (2-tailed)	0.013	0.003	0.022	0.776

*** *p* < 0.01, ** *p* < 0.05, * *p* < 0.1.

**Table 6:** Co-linearity analysis results of the spatial properties variables.

Indicators	In	MD	Con	VC_M_
Tolerance	0.218	0.687	0.201	0.284
VIF	4.590	1.460	4.980	3.520

**Table 7:** Summary of regression models for KD1 (teacher-student conflict).

Models	R^2^	Adjusted R^2^	R^2^ Change	Std. Error of the Estimate
1	^a^ 0.057	0.048	**0.057**	7.502
2	^b^ 0.058	0.040	0.001	9.848
3	^c^ 0.128	0.102	**0.070**	9.611
4 (final)	^d^ 0.147	0.113	0.019	9.788

^a^ Predictors: In. ^b^ Predictors: In, MD. ^c^ Predictors: In, MD, Con. ^d^ Predictors: In, MD, Con, VC_M_.

**Table 8:** Standardized test results of the final model for KD1 (teacher-student conflict).

Indicators	In	MD	Con	VC_M_
beta	10.334 ***	−1.698	−0.066 ***	0.721
t	3.04	−0.59	−3.23	1.51
p	0.003	0.558	0.002	0.134

*** *p* < 0.01.

**Table 9:** Summary of regression models for KD2 (verbal bulling).

Models	R^2^	Adjusted R^2^	R^2^ Change	Std. Error of the Estimate
1	^a^ 0.112	0.103	**0.112**	4.741
2	^b^ 0.134	0.117	0.022	6.133
3	^c^ 0.153	0.128	**0.019**	6.152
4 (final)	^d^ 0.159	0.125	0.006	6.323

^a^ Predictors: In. ^b^ Predictors: In, MD. ^c^ Predictors: In, MD, Con. ^d^ Predictors: In, MD, Con, VC_M_.

**Table 10:** Standardized test results of the final model for KD2 (verbal bulling).

Indicators	In	MD	Con	VC_M_
beta	5.585 **	1.647	−0.023 *	0.257
t	2.54	0.88	−1.69	0.82
*p*	0.013	0.379	0.094	0.413

** *p* < 0.05, * *p* < 0.1.

**Table 11:** Summary of regression models for KD3 (physical conflict).

Models	R^2^	Adjusted R^2^	R^2^ Change	Std. Error of the Estimate
1	^a^ 0.084	0.754	**0.084**	3.572
2	^b^ 0.133	0.117	**0.049**	4.565
3	^c^ 0.209	0.186	**0.076**	4.422
4 (final)	^d^ 0.211	0.179	0.002	4.550

^a^ Predictors: In. ^b^ Predictors: In, MD. ^c^ Predictors: In, MD, Con. ^d^ Predictors: In, MD, Con, VC_M_.

**Table 12:** Standardized test results of the final model for KD3 (physical conflict).

Indicators	In	MD	Con	VC_M_
beta	−6.521 ***	4.616 ***	0.029 ***	−0.100
t	−4.12	3.44	3.00	−0.45
*p*	0.000	0.001	0.003	0.652

*** *p* < 0.01.

**Table 13:** Summary of regression models for KD4 (external intrusion).

Models	R^2^	Adjusted R^2^	R^2^ Change	Std. Error of the Estimate
1	^a^ 0.002	−0.008	0.002	4.429
2	^b^ 0.200	0.185	**0.198**	5.208
3	^c^ 0.349	0.329	**0.149**	4.767
4 (final)	^d^ 0.349	0.323	0.000	4.909

^a^ Predictors: In. ^b^ Predictors: In, MD. ^c^ Predictors: In, MD, Con. ^d^ Predictors: In, MD, Con, VC_M_.

**Table 14:** Standardized test results of the final model for KD4 (external intrusion).

Indicators	In	MD	Con	VC_M_
beta	−6.306 ***	−3.510 **	0.045 ***	−0.034
t	−3.70	−2.42	4.38	−0.14
*p*	0.000	0.017	0.000	0.888

*** *p* < 0.01, ** *p* < 0.05.

**Table 15:** Model checking results of the final model for each type of outdoor school violence.

Indicators		KD1	KD2	KD3	KD4
the difference between R^2^ and adjusted R^2^		0.034	0.034	0.032	0.026
Durbin-Watson		1.162	1.234	1.530	1.625
Cook’s Distance	Mean	0.010	0.012	0.009	0.015
	Minimum	0.000	0.000	0.000	0.000
	Maximum	0.189	0.281	0.075	0.259

## Data Availability

Not applicable.

## Appendix A

**Table A1:** Basic information for tested students based on stratification and random sampling.

Level	Class	Subjects Number/Total Number (Stu.)	Sampling Ratio (%)	Sample Male Number/Sample Female Number (Stu.)	Mean Age
Freshman	1	14/39	35.90	7/7	14.93
	2	13/36	36.11	6/7	14.92
	3	13/38	34.21	6/7	14.85
	4	13/37	35.14	6/7	14.92
	5	13/37	35.14	6/7	15.00
	6	13/38	34.21	7/6	15.00
	7	13/37	35.14	7/6	14.92
	8	14/39	35.90	7/7	14.86
	9	13/37	35.14	7/6	15.00
	10	13/38	34.21	7/6	14.85
Sophomore	1	13/36	36.11	6/7	15.92
	2	12/35	34.29	6/6	15.92
	3	13/37	35.14	6/7	15.85
	4	12/35	34.29	6/6	16.00
	5	13/36	36.11	6/7	15.77
	6	12/34	35.29	6/6	15.92
	7	12/35	34.29	6/6	16.00
	8	13/36	36.11	7/6	15.92
	9	13/37	35.14	7/6	15.85
	10	12/35	34.29	6/6	15.83
	11	12/34	35.29	6/6	16.00
	12	13/37	35.14	7/6	15.85
	13	12/35	34.29	6/6	16.00
Senior	1	14/40	35.00	7/7	16.93
	2	14/40	35.00	7/7	16.86
	3	15/42	35.71	7/8	16.93
	4	13/38	34.21	6/7	16.92

**Table A2:** Questionnaire Details.

Dear Tested Subjects. Hello! Sincerely thank you for taking your valuable time to participate in our questionnaire activities. In order to fully protect your privacy, we guarantee that we will not disclose your personal information (including name, contact details, email address, etc.) in any form.					
**Part One: Personal Information Statistics**					
1. What is your gender?					
A. Male		B. Female			
2. What grade are you currently in?					
A. Senior year	B. Senior year	C. Senior year			
3. Where does your family live?					
A. Rural area	B. Suburban area	C. Urban area			
4. Are you a left-behind child?					
A. Yes		B. No			
**Part Two: Outdoor School Violence Statistics**					
1. During your formative years, were you subjected to or did you inflict violence on others in school? (Please place a check mark in the matching blank box, multiple answers allowed)					
Cynicism from a teacher	( )	Refusal to allow others to participate in group activities			( )
Being physically punished by a teacher	( )	Exclusion of the same or opposite sex			( )
Insulting nicknames	( )	Kicking and punching others			( )
Isolating others	( )	Fighting and brawling			( )
Ridiculing others for being gender non-conforming	( )	Being intimidated or threatened by someone from outside the school			( )
Pushing or shoving someone on purpose	( )	Extortion of property by someone from outside the school			( )
Verbal threats	( )	Fighting with people from outside the school			( )
2. To your knowledge, outdoor school violence at your school generally occurs mainly in (multiple choice)?					
Office entrance	( )	The way to school			( )
Classroom entrance	( )	School entrance			( )
School corridor	( )	Entertainment venues			( )
Playground	( )	Stairwell			( )
Area near the toilet	( )	Secluded grove			( )
Area around the dormitory	( )				
3. To your knowledge, which type of abuser predominates in your school (multiple answers allowed)?					
Male	( )	Female			( )
Senior class	( )	Junior class			( )
Same class	( )	Others (teachers or external personnel)			( )
4. To your knowledge, are there any teacher-student conflicts in your school?					
Yes		No			
5. To your knowledge, please mark on the campus master plan below the spaces where you have experienced or observed outdoor school violence occurring. Please be careful and discreet.					
/					
**Part Three: Current Environmental Assessment**					
1. The following questions ask how you feel about the current outdoor campus environment, please select the level of agreement based on your own experience. Please answer in an objective manner.					
Item	Strongly Agreed	Agreed	Neutral	Disagreed	Strongly Disagreed
Q1: Visual blind spots in landscape space	( )	( )	( )	( )	( )
Q2: Less road lamps or weak light	( )	( )	( )	( )	( )
Q3: Remote dormitory building	( )	( )	( )	( )	( )
Q4: Narrow school entrance and poor traffic order	( )	( )	( )	( )	( )
Q5: Hidden space blind spots on campus	( )	( )	( )	( )	( )
Q6: Lack of leisure facilities in public space	( )	( )	( )	( )	( )
Q7: Distant stadiums	( )	( )	( )	( )	( )
Q8: Low campus fence	( )	( )	( )	( )	( )
Q9: Lack of protective fence in dormitory	( )	( )	( )	( )	( )
Q10: Too dense and chaotic road network	( )	( )	( )	( )	( )
Q11: Confusing planning of school buildings	( )	( )	( )	( )	( )
Q12: Poorly managed green space	( )	( )	( )	( )	( )
Q13: Untimely litter removal	( )	( )	( )	( )	( )
Q14: Lack of security patrols at night	( )	( )	( )	( )	( )
Q15: Lack of security bulletin board	( )	( )	( )	( )	( )
Q16: Public facilities damaged	( )	( )	( )	( )	( )
The questionnaire section has been closed. Your responses will be counted anonymously and confidentiality will be guaranteed during the research process. Thank you very much for your support and cooperation with our team!					

**Table A3:** Perpetrators, victims, bystanders, non-experiencers of different violence types. *N* = 338, female 50.59%.

Violence Types	Violence Activities	Perpetrators (%/Stu.)	Victims (%/Stu.)	Bystanders (%/Stu.)	Non-Experiencers (%/Stu.)
Teacher-student Conflict	Cynicism	4.44/15	23.08/78	37.28/126	35.21/119
	Corporal punishment	6.21/21	21.01/71	38.17/129	34.62/117
Verbal bullying	Insulting nickname	15.98/54	13.02/44	41.42/140	29.59/100
	Isolating others	15.09/51	11.54/39	40.53/137	32.84/111
	Ridiculing others for the incompatibility of their personality with their gender	7.10/24	5.92/20	42.90/145	44.08/149
	Deliberately pushing others	7.99/27	4.44/15	44.08/149	43.49/147
	Verbal threat	9.47/32	9.47/32	43.49/147	37.57/127
	Refusing others to participate in collective activities	7.10/24	4.44/15	43.49/147	44.97/152
	Reject people of the same gender or opposite gender	8.58/29	5.92/20	42.01/142	43.49/147
Physical conflict	Kicking and beating	17.75/60	8.58/29	33.73/114	39.94/135
	Fighting	6.67/23	5.33/18	52.89/178	35.21/119
External intrusion	Intimidation and threat	1.48/5	9.17/31	39.94/135	46.15/156
	Blackmailing	3.25/11	5.33/18	39.94/135	51.48/174
	Affray	0.59/2	2.66/9	27.51/93	69.23/234

**Table A4:** Evaluation results of tested students on the outdoor campus environment. *N* = 338, female 50.59%.

Item	Strongly Agreed (%/Stu.)	Agreed (%/Stu.)	Neutral (%/Stu.)	Disagreed (%/Stu.)	Strongly Disagreed (%/Stu.)
Q1: Visual blind spots in landscape space	7.99/27	11.24/38	52.07/176	13.31/45	15.38/52
Q2: Less road lamps or weak light	5.03/17	12.13/41	45.56/154	17.75/60	19.53/66
Q3: Remote dormitory building	6.80/23	11.54/39	45.27/153	16.86/57	19.53/66
Q4: Narrow school entrance and poor traffic order	13.02/44	15.98/54	43.49/147	13.31/45	14.20/48
Q5: Hidden space blind spots on campus	5.92/20	13.02/44	42.60/144	21.60/73	16.86/57
Q6: Lack of leisure facilities in public space	5.03/17	12.43/42	42.60/144	19.53/66	20.41/69
Q7: Distant stadiums	8.58/29	11.54/39	42.01/142	17.16/58	17.75/60
Q8: Low campus fence	0.89/3	8.88/30	39.05/132	21.89/74	29.29/99
Q9: Lack of protective fence in dormitory	1.78/6	11.24/38	38.76/131	23.37/79	24.85/84
Q10: Too dense and chaotic road network	2.66/9	8.58/29	43.49/147	18.64/63	26.63/90
Q11: Confusing planning of school buildings	4.14/14	6.80/23	47.34/160	21.60/73	20.12/68
Q12: Poorly managed green space	0.89/3	5.33/18	41.72/141	21.30/72	30.77/104
Q13: Untimely litter removal	1.48/5	5.03/17	38.46/130	22.49/76	32.54/110
Q14: Lack of security patrols at night	4.73/16	10.65/36	42.90/145	21.30/72	20.41/69
Q15: Lack of security bulletin board	4.44/15	11.24/38	46.45/157	19.23/65	18.64/63
Q16: Public facilities damaged	1.78/6	9.76/33	45.86/155	18.64/63	23.96/81

## Appendix B

The questionnaire part “Current Environmental Assessment” consisted of 16 items, and its reliability and validity tests were carried out in IBM SPSS Statistics 26.

Firstly, the internal consistency was tested for reliability using Cronbach’s alpha, Spearman–Brown coefficient, and McDonalds omega. The calculation results showed that Cronbach’s alpha for the questionnaire was 0.922, while the McDonald’s omega was 0.934. When the 16 items were analyzed in half, the Spearman–Brown coefficient was 0.905. As can be seen, the results for the three important indicators were all greater than 0.900, which illustrated that they were all within good reliability indicators according to the relevant references [110,111], suggesting that the reliability quality of the study data can be affirmed.

Secondly, the structural validity was tested using exploratory factor analysis, containing KMO, Bartlett’s sphericity, variance explained, and commonality, with the difference considered statistically significant at *p* < 0.05. With all items subjected to exploratory factor analysis simultaneously, the commonality value for Q4 was found to be less than 0.4, indicating a very weak relationship between the factors and it. After excluding Q4 [112], exploratory factor analysis was performed again and the results presented the KMO of 0.933 (greater than 0.8, showing a good fit for factor analysis) as well as the data passing the Bartlett’s sphericity test (*p* < 0.05), satisfying the prerequisite requirements, implying that the data could be used for factor analysis studies. In addition, two factors (both with Eigenroot values greater than 1) were extracted, which led to a rotated variance explained of 21.473% and 36.377%, respectively, with a cumulative variance explained by the rotation of 57.850%. Table A5 displays the classification of the two factors and their factor loadings and commonality, with Factor1 containing Q1, Q2, Q3, and Q11, and Factor2 containing Q5, Q6, Q7, Q8, Q9, Q10, Q12, Q13, Q14, Q15, and Q16. The communality value for each item was above 0.4, signifying a strong correlation between the items and the factors. It can be believed that all of the above indicators presented that the questionnaire had good construct validity [110].

**Table A5:** Factor loadings and commonality of the 15 items of the questionnaire part “Current Environmental Assessment”.

Item	Factor Loading		Communality
	Factor 1	Factor 2	
Q1: Visual blind spots in landscape space		0.720	0.530
Q2: Less road lamps or weak light		0.797	0.691
Q3: Remote dormitory building		0.766	0.631
Q5: Hidden space blind spots on campus	0.500		0.480
Q6: Lack of leisure facilities in public space	0.689		0.489
Q7: Distant stadiums	0.640		0.460
Q8: Low campus fence	0.779		0.692
Q9: Lack of protective fence in dormitory	0.774		0.611
Q10: Too dense and chaotic road network	0.668		0.522
Q11: Confusing planning of school buildings		0.700	0.565
Q12: Poorly managed green space	0.750		0.656
Q13: Untimely litter removal	0.788		0.654
Q14: Lack of security patrols at night	0.498		0.416
Q15: Lack of security bulletin board	0.655		0.585
Q16: Public facilities damaged	0.790		0.696

Note: The commonality greater than 0.40 indicates that there is a strong correlation between the items and the factors.

Thirdly, validation factor analysis was carried out for a total of two factors, and 15 items, containing three indicators, average variance extracted (AVE), construct reliability (CR), and the AVE square root. The effective sample size for this analysis was 338, which exceeded the number of items analyzed. The results revealed that the two factors corresponding to AVE values all greater than 0.5 (Factor 1: 0.502, Factor 2: 0.511) and CR values all above 0.7 (Factor 1: 0.799, Factor 2: 0.919), implying good convergent validity [110,111]. Additionally, the AVE square root values for Factor 1 and Factor 2 were 0.709 and 0.715, respectively, which were greater than the maximum value of 0.590 for the absolute value of the inter-factor correlation coefficient, signifying good discriminant validity [110].

In summary, the reliability and validity of the responses to the scale questions of the questionnaire were validated.

## References

[1] UNESCO (2017). School Violence and Bullying: Global Status Report.

[2] Baker-Henningham H., Bowers M., Francis T., Vera-Hernández M., Walker S.P. (2021). The Irie Classroom Toolbox, a universal violence-prevention teacher-training programme, in Jamaican preschools: A single-blind, cluster-randomised controlled trial. Lancet Glob. Health.

[3] Riehm K.E., Mojtabai R., Adams L.B., Krueger E.A., Mattingly D.T., Nestadt P.S., Leventhal A.M. (2021). Adolescents’ Concerns About School Violence or Shootings and Association With Depressive, Anxiety, and Panic Symptoms. JAMA Netw. Open.

[4] Xu Z., Fang C. (2021). The Relationship between School Bullying and Subjective Well-Being: The Mediating Effect of School Belonging. Front. Psychol..

[5] Bochaver A. (2021). School Bullying Experience and Current Well-Being among Students. Psikhologicheskaya Nauka I Obraz..

[6] Ferrara P., Franceschini G., Namazova-Baranova L., Vural M., Mestrovic J., Nigri L., Giardino I., Pop T.L., Sacco M., Pettoello-Mantovani M. (2019). Lifelong Negative Influence of School Violence on Children. J. Pediatr..

[7] Luo X., Zheng R., Xiao P., Xie X., Liu Q., Zhu K., Wu X., Xiang Z., Song R. (2022). Relationship between school bullying and mental health status of adolescent students in China: A nationwide cross-sectional study. Asian J. Psychiatry.

[8] UNESCO (2018). School Violence and Bullying: Global Status and Trends, Drivers and Consequences.

[9] Ministry of Education, PRC National Statistical Bulletin on the Development of Education in 2017. http://www.moe.gov.cn/jyb_sjzl/sjzl_fztjgb/201807/t20180719_343508.html.

[10] Fu W., Zhou W., Li W., Chen A. Survey Claims 32.4% Incidence of Bullying in Schools: Vulnerable Groups More Likely to Be Bullied. https://share.gmw.cn/edu/2021-11/01/content_1302660247.htm.

[11] Soonjung K., Hayoung K. (2021). Exploring the Educational Possibility of Preventing School Violence by Cultural Transition: Reflecting upon school violence through the objectification phenomenon and revisiting the value of peace, human right, and relationship. J. Korean Educ..

[12] Ilona T., Saulius S., Aurelijus Z., Aleksandras A. (2021). Examining the Relations among Extraversion, Neuroticism, and School Bullying among Lithuanian Adolescents. Eur. J. Contemp. Educ..

[13] Zhang Y., Li Z., Tan Y., Zhang X., Zhao Q., Chen X. (2021). The Influence of Personality Traits on School Bullying: A Moderated Mediation Model. Front. Psychol..

[14] Romeiro J.S., Corrêa M.M., Pazó R., Leite F.M.C., Cade N.V. (2021). Physical violence and associated factors in participants of the National Student Health Survey (NSHS). Cienc. Saude Coletiva.

[15] Buhaș C.L., Judea-Pusta C., Buhaș B.A., Bungau S., Judea A.S., Sava C., Popa V.C., Cioca G., Tit D.M. (2021). Physical, Psychological and Sexual Abuse of the Minor in the Families from the Northwestern Region of Romania- Social and Medical Forensics. Iran. J. Public Health.

[16] Hultin H., Ferrer-Wreder L., Engström K., Andersson F., Galanti M.R. (2021). The Importance of Pedagogical and Social School Climate to Bullying: A Cross-Sectional Multilevel Study of 94 Swedish Schools. J. Sch. Health.

[17] Reisen A., Leite F.M.C., Neto E.T.D.S. (2021). Association between social capital and bullying among adolescents aged between 15 and 19: Relations between the school and social environment. Cienc. Saude Coletiva.

[18] Duque E., Carbonell S., de Botton L., Roca-Campos E. (2021). Creating Learning Environments Free of Violence in Special Education Through the Dialogic Model of Prevention and Resolution of Conflicts. Front. Psychol..

[19] Shik J.H. (2021). A Drama Therapy Program to Improve Spatial Awareness for Treating School Violence: Focusing on the Fairy Tale <The Ugly Duckling>. J. Drama Art Ther..

[20] Oscós-Sánchez M., Lesser J., Oscós-Flores L.D., Pineda D., Araujo Y., Franklin B., Hernández J.A., Hernández S., Vidales A. (2020). The Effects of Two Community-Based Participatory Action Research Programs on Violence Outside of and in School Among Adolescents and Young Adults in a Latino Community. J. Adolesc. Health.

[21] Glubwila S., Sripa K., Thummaphan P. (2021). The model of collaboration integration for preventing and solving the problem of youth violence in educational settings. Curr. Psychol..

[22] Zeng M., Sun W., Mao Y., Liang Y., Wu Q. (2018). Study and enlightenment on prevention measures of overseas campus crime. Urban Des..

[23] Foody M., Challenor L., Murphyy H., Norman J.O. (2018). The Anti-Bullying Procedures for Primary and Post-Primary Schools in Ireland: What Has Been Achieved and What Needs to be done. Bild. Und Erzieh..

[24] Kalichman S.C., Brosig C.L. (1992). Mandatory Child Abuse Reporting Laws: Issues and Implications for Policy. Law Policy.

[25] Kim N. (2015). The Police Action against School Violence in Korea. Ph.D. Thesis.

[26] Sairanen L., Pfeffer K. (2011). Self-reported handling of bullying among junior high school teachers in Finland. Sch. Psychol. Int..

[27] Kim Y.O. (2021). A Study on the Regional Professional Program for the School Violence Prevention. Korean Assoc. Police Sci. Rev..

[28] Sullivan T.N., Farrell A.D., Sutherland K.S., Behrhorst K.L., Garthe R.C., Greene A. (2021). Evaluation of the Olweus Bullying Prevention Program in US Urban Middle Schools Using a Multiple Baseline Experimental Design. Prev. Sci..

[29] Bradshaw C.P., Waasdorp T.E., Debnam K.J., Johnson S.L. (2014). Measuring school climate in high schools: A focus on safety, engagement, and the environment. J. Sch. Health.

[30] Lamoreaux D.J., Sulkowski M.L. (2021). Crime Prevention through Environmental Design in schools: Students’ perceptions of safety and psychological comfort. Psychol. Sch..

[31] Lu P., Chen D., Li Y., Wang X., Yu S. (2022). Agent-Based Model of Mass Campus Shooting: Comparing Hiding and Moving of Civilians. IEEE Trans. Comput. Soc. Syst..

[32] Aggarwal J., Eitland E.S., Gonzalez L.N., Greenberg P., Kaplun E., Sahili S. (2021). Built Environment Attributes and Preparedness for Potential Gun Violence at Secondary Schools. J. Environ. Health.

[33] Ma N., Ma S., Li S., Ma S., Pan X., Sun G. (2021). The Study of Spatial Safety and Social Psychological Health Features of Deaf Children and Children with an Intellectual Disability in the Public School Environment Based on the Visual Access and Exposure (VAE) Model. Int. J. Environ. Res. Public Health.

[34] Cozens P.M., Saville G., Hillier D. (2005). Crime prevention through environmental design (CPTED): A review and modern bibliography. Prop. Manag..

[35] Armitage R. (2018). Burglars’ take on crime prevention through environmental design (CPTED): Reconsidering the relevance from an offender perspective. Secur. J..

[36] Mao Y., Yin L., Zeng M., Ding J., Song Y. (2021). Review of Empirical Studies on Relationship between Street Environment and Crime. J. Plan. Lit..

[37] Tuba K., Colpan E.N. (2022). Design Guidelines for Urban Safety. J. Plan..

[38] Jacobs J. (1961). The Death and Life of Great American Cities.

[39] Jeffery C.R. (1971). Crime Prevention through Environmental Design.

[40] Newman O. (1972). Defensible Space: Crime Prevention through Urban Design.

[41] Cozens P., Love T. (2015). A Review and Current Status of Crime Prevention through Environmental Design (CPTED). J. Plan. Lit..

[42] Liu C. (2014). Environment Redesigned for Crime Prevention with CPTED Strategies: A Case Study of Typical Chinese Community. Appl. Mech. Mater..

[43] Rupp L.A., Zimmerman M.A., Sly K.W., Reischl T.M., Thulin E.J., Wyatt T.A., Stock J. (2020). Community-Engaged Neighborhood Revitalization and Empowerment: Busy Streets Theory in Action. Am. J. Community Psychol..

[44] Curtis-Ham S., Cantal C., Gravitas Research Ltd (2022). Locks, lights, and lines of sight: An RCT evaluating the impact of a CPTED intervention on repeat burglary victimisation. J. Exp. Criminol..

[45] Surette R., Stephenson M. (2019). Expectations versus effects regarding police surveillance cameras in a municipal park. Crime Prev. Community Saf..

[46] Cho M.-G., Park C., Jang J.-I. (2018). The Effects of Urban Park and Vegetation on Crime in Seoul and Its Planning Implication to CPTED. J. Korean Inst. Landsc. Archit..

[47] Yeom S.-J., Hong Y.-S. (2017). A Case Study on Application of CPTED of Park Development Guidelines. J. Environ. Sci. Int..

[48] Zhang Y., Yang F., Wang M. (2022). Investigation of Urban Park Crime Prevention Based on CPTED Theory and Space Syntax Taking People’s Park and Zijingshan Park in Zhengzhou as Examples. J. Southwest Univ. Nat. Sci. Ed..

[49] Su J., Yin W., Zhang R., Zhao X., Chinkam O.N., Zhang H., Li F., Kang L. (2021). A Multi-source Data Based Analysis Framework for Urban Greenway Safety. Teh. Vjesn.-Tech. Gaz..

[50] Erman A. (2021). Evaluation of Crime Prevention Theories through Environmental Design in Urban Renewal: A Case Study of Ankara—The Vicinity of Haci Bayram Mosque. ICONARP Int. J. Archit. Plan..

[51] Kang H. (2022). Effect of the Local Community’s Perception of Urban Regeneration and CPTED on Safety Life Satisfaction. Korean Secur. J..

[52] Yang M.-Y., Cho J.-H. (2021). A Study of CPTED Design Color to Urban Regeneration in Alley Street of Waterfront—Focused on Bongsan Village, Bongrae 2-dong, Yeongdo-gu, Busan. J. Korea Soc. Color Stud..

[53] Badiora A.I., Dada O.T., Adebara T.M. (2021). Correlates of crime and environmental design in a Nigerian international tourist attraction site. J. Outdoor Recreat. Tour..

[54] Sun Y.N. (2017). The Research on the Design Method of Crime Prevention in Hospital Buildings. Master’s Thesis.

[55] El-Hadedy N., El-Husseiny M. (2021). Evidence-Based Design for Workplace Violence Prevention in Emergency Departments Utilizing CPTED and Space Syntax Analyses. HERD.

[56] Hyeji R. (2021). A Study on the Physical Environmental Factors Causing Crime Fear of College Towns in Urban and Rural Complex Areas—Focusing on Hongseong-eup, Hongseong-gun. J. Korea Intitute Spat. Des..

[57] Jones F., Sloboden J. (2017). Jacksonville, Florida, Transportation Authority’s Mobility Corridors: Improving Transit System Performance through Enhanced Safety and Urban Design. Transp. Res. Rec..

[58] Kim W. (2021). A Study on the Effects of CPTED on Social Interaction among Students—Focused on the Environment of University Dormitory. J. Archit. Inst. Korea.

[59] He L., Kwan S.-H. (2022). A Study on the 3rd Generation CPTED Process through Double Diamond. J. Korea Contents Assoc..

[60] Gouveia F., Sani A., Guerreiro M., Azevedo V., Santos H., Nunes L.M. (2021). Mapping CPTED parameters with the LookCrim application. Crime Prev. Community Saf..

[61] Azevedo V., Maia R.L., Guerreiro M.J., Oliveira G., Sani A., Caridade S., Dinis M.A.P., Estrada R., Paulo D., Magalhães M. (2021). Looking at crime-communities and physical spaces: A curated dataset. Data Brief.

[62] Wilcox P., Quisenberry N., Jones S. (2003). The Built Environment and Community Crime Risk Interpretation. J. Res. Crime Delinq..

[63] Johnson S.D., Bowers K.J. (2010). Permeability and Burglary Risk: Are Cul-de-Sacs Safer?. J. Quant. Criminol..

[64] Feng J., Huang L., Dong Y., Song L. (2012). Research on the spatia-temporal characteristics and mechanism of urban crime:A case study of property crime in Beijing. Acta Geogr. Sin..

[65] He R., Xu Y., Jiang S. (2022). Applications of GIS in Public Security Agencies in China. Asian J. Criminol..

[66] Leydesdorff L. (2008). Patent classifications as indicators of intellectual organization. J. Am. Soc. Inf. Sci. Technol..

[67] Mehrotra D., Kumar M., Singh S.N., Sukhija K. (2022). Spatial and temporal trends reveal: Hotspot identification of crimes using machine learning approach. Int. J. Comput. Sci. Eng..

[68] Abd El AZIZ N.A. (2020). Space Syntax as a Tool to Measure Safety in Small Urban Parks: A Case Study of Rod El Farag Park in Cairo, Egypt. Landsc. Archit. Front..

[69] Matijosaitiene I. (2016). Combination of CPTED and space syntax for the analysis of crime. Saf. Communities.

[70] Hillier B., Hanson J. (1984). The Social Logic of Space.

[71] Sherman S.A. (2022). Policing the Campus: Police Communications and near-Campus Development across Atlanta’s University Communities. Plan. Theory Pract..

[72] Ministry of Education, PRC National Statistical Bulletin on the Development of Education in 2011. http://www.moe.gov.cn/srcsite/A03/s180/moe_633/201208/t20120830_141305.html.

[73] Li H.M. (2018). A Study on the Strategy of Prevention and Control of Bullying in the Rural Boarding Schools: Case of S Township Middle School in Henan Province. Master’s Thesis.

[74] William G.C. (1977). Sampling Techniques.

[75] Huang D. (2020). Perceived Safety Evaluation and Design Strategy of Guangzhou Higher Education Mega Center Based on Crime Prevention through Environmental Design. Master’s Thesis.

[76] Liu H., Zhang Z., Ma X., Lu W., Li D., Kojima S. (2021). Optimization Analysis of the Residential Window-to-Wall Ratio Based on Numerical Calculation of Energy Consumption in the Hot-Summer and Cold-Winter Zone of China. Sustainability.

[77] Gopalakrishnan R., Guevara C.A., Ben-Akiva M. (2020). Combining multiple imputation and control function methods to deal with missing data and endogeneity in discrete-choice models. Transp. Res. Part B Methodol..

[78] Wang P. (2021). A Survey of Bullying in Rural Junior High Schools: From the Perspective of Students. Master’s Thesis.

[79] Wamg H. (2020). Research on the Status and Prevention of School Bullying in Rural Junior Middle School. Master’s Thesis.

[80] Jiaping Y. (2020). The Current Situation and Countermeasures of Bullying in Private Boarding Junior Middle Schools from the Perspective of Social Ecosystem: Taking a Middle School. Master’s Thesis.

[81] López-Castedo A., García D., José D.A., Roales E.A. (2018). Expressions of school violence in adolescence. Psicothema.

[82] Kearney C.A. (2008). School absenteeism and school refusal behavior in youth: A contemporary review. Clin. Psychol. Rev..

[83] Cheng J., Sun L., Wang T., Xu F., Sun L. (2021). The coupling relationship between crime distribution and urban environment in Lanzhou city center. Sci. Surv. Mapp..

[84] Hillier B. (1996). Space is the Machine: A Configurationally Theory of Architecture.

[85] Alalouch C., Al-Hajri S., Naser A., Al Hinai A. (2019). The Impact of Space Syntax Spatial Attributes on Urban Land Use in Muscat: Implications for Urban Sustainability. Sustain. Cities Soc..

[86] Field A. (2009). Discovering Statistics Using SPSS.

[87] Bowerman B.L., O’Connell R.T. (2000). Linear Statistical Models: An Applied Approach.

[88] Ralph J. (2012). Linear Statistical Models: An Applied Approach. Technometrics.

[89] Chatterjee S., Hadi A.S. (2006). Regression Analysis by Example.

[90] Wang M. (2013). Research on Web-Based Spatial Data Grab and Evaluation. Ph.D. Thesis.

[91] Weisberg S. (2005). Applied Linear Regression.

[92] Moon B., McCluskey J. (2016). School-Based Victimization of Teachers in Korea: Focusing on Individual and School Characteristics. J. Interpers. Violence.

[93] Alves A.G., Cesar F.C.R., Barbosa M.A., Oliveira L.M.D.A.C., da Silva E.A.S., Rodríguez-Martín D. (2022). Dimensions of student violence against the teacher. Cienc. Saude Coletiva.

[94] Jia Y. (2021). Research on the Crime Prevention of Aggressive School Violence Based on CPTED Theory. Master’s Thesis.

[95] Kweon J. (2018). A Study on the Architectural Planning of Elementary School for Violence Prevention—Through Analyzing Space Composition and Visibility Characteristics of Elementary Schools in Daegu City. J. Reg. Assoc. Archit. Inst. Korea.

[96] Ju M.-O., Lee C.-H. (2017). School Territories and Complementary Strategies of CPTED for Each School Territory. J. Community Saf. Secur. Environ. Des..

[97] Charles C.L. (2022). Collective efficacy and the built environment. Criminology.

[98] Jiang D., Qiu B. (2020). Comparison of effects of spatial anticrime in open communities in China. J. Asian Arch. Build. Eng..

[99] Hu H., Huang X., Suhaim M.A., Zhang H. (2021). Comparison of compression estimations under the penalty functions of different violent crimes on campus through deep learning and linear spatial autoregressive models. Appl. Math. Nonlinear Sci..

[100] Qiao Y., Xing Y., Duan J., Bai C., Pan Y., Cui Y., Kong J. (2010). Prevalence and associated factors of school physical violence behaviors among middle school students in Beijing. Chin. J. Epidemiol..

[101] Wang H., Tang J., Dill S.-E., Xiao J., Boswell M., Cousineau C., Rozelle S. (2022). Bullying Victims in Rural Primary Schools: Prevalence, Correlates, and Consequences. Int. J. Environ. Res. Public Health.

[102] Sadjadi M., Blanchard L., Brülle R., Bonell C. (2021). Barriers and facilitators to the implementation of Health-Promoting School programmes targeting bullying and violence: A systematic review. Health Educ. Res..

[103] Evans D., Butterworth R., Law G.U. (2019). Understanding associations between perceptions of student behaviour, conflict representations in the teacher-student relationship and teachers’ emotional experiences. Teach. Teach. Educ..

[104] Scharpf F., Kirika A., Masath F.B., Mkinga G., Ssenyonga J., Nyarko-Tetteh E., Nkuba M., Karikari A.K., Hecker T. (2021). Reducing physical and emotional violence by teachers using the intervention Interaction Competencies with Children—For Teachers (ICC-T): Study protocol of a multi-country cluster randomized controlled trial in Ghana, Tanzania, and Uganda. BMC Public Health.

[105] Hyoen K.N., JihooA K. (2016). Study on Quantitative CPTED Guideline Selection Model for Public Street Considering Spatial Characteristics of Juvenile Crimes in School Surroundings. J. Digit. Des..

[106] Wahab A.A., Sakip S.R.M. (2020). Mapping Isolated Places in School in Concurrence with Bullying Possibility Elements. Environ. Behav. Proc. J..

[107] Shariati A. (2021). Crime prevention through environmental design (CPTED) and its potential for campus safety: A qualitative study. Secur. J..

[108] Tomita M., Tago T., Ohuchi H. (2003). Correlation between Color Composition of District, Environment Recognition and Behavioral Characteristics in Cityscape: Case study in Ginza and Harajuku area (Environmental Engineering). AIJ J. Technol. Des..

[109] Braun C.C., Greeno B., Silver N.C. (1994). Differences in Behavioral Compliance as a Function of Warning Color. Proc. Hum. Factors Ergon. Soc. Annu. Meet..

[110] Ross S. (2019). Introduction to Probability Models.

[111] Chung R.H., Kim B.S., Abreu J.M. (2004). Asian American multidimensional acculturation scale: Development, factor analysis, reliability, and validity. Cult. Divers. Ethn. Minor. Psychol..

[112] Yoon E., Langrehr K., Ong L.Z. (2010). Content Analysis of Acculturation Research in Counseling and Counseling Psychology: A 22-Year Review. J. Couns. Psychol..

